# Sabotaged Integral HSC Heterogeneity Underlies Essential Thrombocythemia Development

**DOI:** 10.1002/advs.202505249

**Published:** 2025-11-21

**Authors:** Jingyuan Tong, Di Wang, Haoze Song, Ting Sun, Yanhong Zhao, Wenjing Gu, Lexuan Lin, Yitong Zhao, Yipeng Liang, Xu Jin, Rongfeng Fu, Mankai Ju, Jie Gao, Jinfa Ma, Chengjie Gao, Anqi Zhang, Zhijian Xiao, Erlie Jiang, Renchi Yang, Shihui Ma, Lei Zhang, Lihong Shi

**Affiliations:** ^1^ State Key Laboratory of Experimental Hematology National Clinical Research Center for Blood Diseases Haihe Laboratory of Cell Ecosystem Institute of Hematology & Blood Diseases Hospital Chinese Academy of Medical Sciences & Peking Union Medical College Tianjin 300020 China; ^2^ CAMS Key Laboratory of Gene Therapy for Blood Diseases Tianjin Key Laboratory of Gene Therapy for Blood Diseases Tianjin 300020 China; ^3^ Tianjin Institutes of Health Science Tianjin 301600 China

**Keywords:** CALR, CXCR4, essential thrombocythemia (ET), MPL, myeloproliferative neoplasms (MPN), scRNA‐seq, triple negative ET

## Abstract

Essential thrombocythemia (ET) includes the *JAK2* ‐, *CALR*‐, and *MPL*‐mutated subtypes, and a triple‐negative (TN) ET subtype, which lacks these canonical drivers. How specific driver mutations affect hematopoietic stem cell (HSC) heterogeneity and their relation to ET pathogenesis remain unclear. Here, by using single‐cell RNA sequencing (scRNA‐seq) combined with driver mutation detection across ET patients, it is found that *MPL*‐mutated HSCs exhibited aberrant metabolism, *CALR*‐mutated HSCs displayed active cell cycling, while *JAK2* ^V617F^‐mutated HSCs demonstrated enhanced megakaryocyte (Mk) priming capacity and interferon (IFN) response. An HSC subset is identified in TN ET that transcriptionally resembled driver‐mutated HSCs, exhibiting enhanced megakaryocytic priming and proliferative activity. Notably, the frequency of a CXCR4^+^ HSC subset is reduced across the ET spectrum. Loss of CXCR4^+^ HSCs skewed lineage differentiation of HSCs toward the myeloid lineage, whereas restoring this subset delayed the onset of ET. Altogether, this study reveals both the shared and distinct molecular features of mutant HSCs in ET and provides novel insights into the pathogenesis and potential therapeutic strategies of ET.

## Graphical abstract

Single‐cell RNA sequencing (scRNA‐seq) maps how distinct driver mutations remodel hematopoietic stem cell (HSC) programs across essential thrombocythemia (ET). Comparative analysis uncovers both shared and subtype‐specific molecular signatures, identifies a triple‐negative (TN)‐associated HSC population enriched with malignant traits, and reveals the loss of CXCR4⁺ HSCs as a convergent mechanism contributing to abnormal lineage commitment and disease progression.

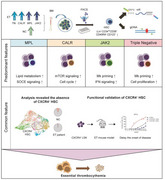

## 1 Introduction

Philadelphia‐negative myeloproliferative neoplasms (Ph^−^ MPNs) are clonal disorders driven by malignant hematopoietic stem cells (HSCs),^[^ ^1^, ^2^ ^]^ comprising polycythemia vera (PV), essential thrombocythemia (ET), and primary myelofibrosis (PMF).^[^ ^3^ ^]^ Among them, ET is associated with increased megakaryocyte (Mk) and platelet production in the bone marrow (BM), with most cases associated with the janus kinase 2 gene (*JAK2*),^[^ ^3^, ^4^, ^5^ ^]^ calreticulin (*CALR)*,^[^ ^6^, ^7^ ^]^ or thrombopoietin receptor (*TPOR/MPL)* ^[^ ^8^, ^9^, ^10^ ^]^ driver mutations.^[^ ^11^, ^12^, ^13^ ^]^ However, ≈12% of ET patients lack these driver mutations and are classified as the triple‐negative (TN) ET subtype.^[^ ^14^ ^]^ Despite their genetic heterogeneity, constitutive activation of the JAK‐signal transducer and activator of transcription (STAT) signaling pathway is regarded as a common pathogenic mechanism across ET subtypes^[^ ^15^ ^]^ that contributes to their overlapping clinical phenotypes. Nevertheless, prognosis, disease progression, and the risk of leukemic transformation vary across the ET spectrum, while the underlying cellular and molecular mechanisms remain largely unknown.

Understanding how specific mutations alter HSC behavior is critical for elucidating the pathogenesis of ET. Given the coexistence of mutant and non‐neoplastic cells in the BM of MPN patients, distinguishing mutated subpopulations and characterizing their specific transcriptional changes at the single‐cell level is essential. Our previous studies and those of others^[^ ^16^, ^17^, ^18^, ^19^ ^]^ have employed single‐cell transcriptomics with mutation detection to discover the specific characteristics of mutant cells. For instance, *JAK2* ^V617F^‐mutated HSCs in ET exhibit increased Mk priming and elevated interferon (IFN) responses; while in MF, *JAK2* ^V617F^‐mutated HSPCs display pro‐inflammatory signatures within HSCs and profibrotic inflammatory characteristics in Mk progenitors (MkPs),^[^ ^16^, ^17^, ^18^, ^20^, ^21^, ^22^ ^]^ supporting a mutation‐driven reprogramming of stem and progenitor cells.

In *CALR*‐mutated ET, mutant CALR aberrantly binds MPL or weakly interacts with granulocyte‐colony stimulating factor receptor (G‐CSFR), leading to downstream activation of JAK‐STAT3/STAT5 and MAPK signaling.^[^ ^6^, ^23^, ^24^, ^25^, ^26^, ^27^ ^]^ Single‐cell transcriptomic studies of ET patients have revealed that *CALR*‐mutated BM cells display higher proliferation and unfolded protein response (UPR) than non‐mutated hematopoietic stem/progenitor cells (HSPCs) and Mks.^[^ ^19^, ^28^ ^]^ Moreover, a knock‐in mouse model of ET with homozygous *CALR*‐mutated HSCs exhibits increased cholesterol biosynthesis as compared to their wildtype (WT) counterparts.^[^ ^29^ ^]^ However, most ET patients harbor heterozygous *CALR* mutations, the transcriptomic changes in highly purified populations of *CALR*‐mutated HSCs from ET patients are still unclear. Additionally, two types of *CALR* mutations, namely type 1 (52‐bp deletion) and type 2 (5‐bp insertion),^[^ ^6^, ^7^ ^]^ are usually associated with different clinical outcomes. ET patients with type 2 *CALR* mutations typically exhibit higher platelet counts and better response to IFNα treatment.^[^ ^7^, ^21^, ^30^ ^]^ Yet, the cellular mechanisms underlying these differences remain unclear.

Although *MPL* mutations occur in a small subset (1–4%) of ET patients,^[^ ^8^ ^]^ they remain of particular interest due to their association with more aggressive clinical phenotypes and poorer prognosis. As MPL is expressed in MkPs, Mks, and platelets,^[^ ^31^ ^]^ its gain‐of‐function mutations greatly fuel the production of Mks and platelets. Mechanistically, these mutations stabilize the MPL homodimer, activating the downstream JAK/STAT, RAS/MAPK, and PI3K pathways.^[^ ^32^ ^]^ Additionally, MPL serves as the only hematopoietic growth factor receptor expressed by HSCs.^[^ ^33^ ^]^ Moreover, the TPO/MPL signaling axis plays crucial roles in HSC quiescence.^[^ ^34^ ^]^ Indeed, *Mpl* ^−/−^ mice exhibit reduced BM HSCs, multilineage progenitors, and competitive repopulating capacity.^[^ ^33^, ^35^, ^36^ ^]^ Despite these important findings, the understanding of molecular changes in *MPL*‐mutated HSCs in ET remains limited, largely due to a lack of direct methods for accurately characterizing mutant HSCs.

Extensive efforts have been made to identify the potential driver mutations in patients with TN ET using high‐throughput genetic sequencing. These endeavors have revealed additional non‐canonical mutations in *JAK2*, *MPL*, and *CALR* genes, along with mutations in *TET2*, *KIT*, and *RUNX1*.^[^ ^14^, ^37^, ^38^ ^]^ In many patients with TN ET, however, the genetic drivers and/or the exact roles of the identified mutations are unclear; thus, the pathogenesis of TN ET remains an enigma. Given that ET development may be sustained by heterogeneous HSC subsets,^[^ ^16^, ^18^, ^19^ ^]^ it is plausible that specific transcriptionally primed HSC populations might contribute to TN ET pathogenesis.

In this study, we performed a single‐cell transcriptomic analysis, coupled with single‐cell driver mutation detection, using BM‐derived HSCs from ET patients harboring *JAK2* ^V617F^, *CALR*, or *MPL* mutations, and those with TN ET. Our main aim was to investigate the common and unique molecular features of HSCs across ET spectrum, thereby providing insights into disease mechanisms, informing diagnostic strategies and guiding precision therapies.

## 2 Results

### 2.1 Distinguishing Mutated and Non‐Mutated HSCs in the BM of ET Patients Carrying Distinct Driver Mutations

**Figure 1:**
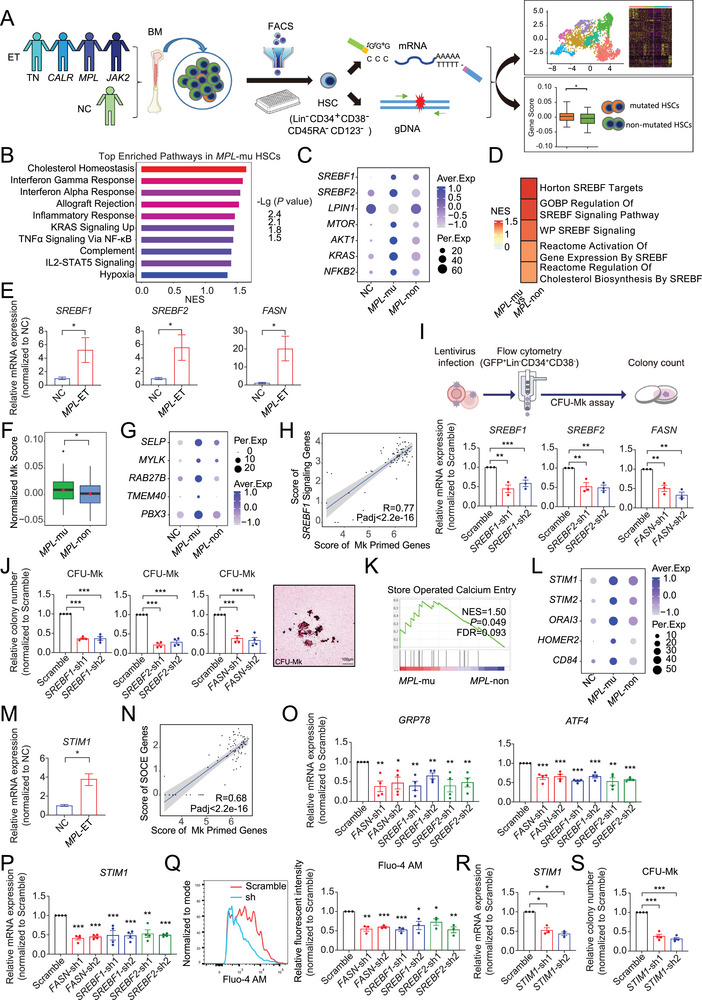
*MPL*‐mutated HSCs in ET are characterized by prominent metabolic disturbances. A) Schematic of the experimental design. In brief, fluorescence‐activated cell sorting (FACS)‐purified hematopoietic stem cells (HSCs) (Lin^−^CD34^+^CD38^−^CD45RA^−^CD123^−^) from normal controls (NCs) (*n* = 15) and untreated *JAK2* ^V617F^ ‐mutated (*n* = 7), *CALR*‐mutated (*n* = 13), *MPL*‐mutated (*n* = 10), and triple‐negative (TN) (*n* = 5) essential thrombocythemia (ET) patients, were subjected to scRNA‐seq. Here, we applied the modified 3′‐TARGET‐seq method, in which transcriptome and driver genetic mutation are detected in parallel. Finally, transcriptional information and mutational type (including mutated and non‐mutated) of single HSCs were integrated and analyzed. B) Bar plot displaying the top significantly enriched pathways identified by Gene Set Enrichment Analysis (GSEA) in *MPL*‐mutated (*MPL‐*mu) versus non‐mutated (*MPL*‐non) HSCs from patients with ET. C) Dot plot displaying the expression of key upstream regulators of *SREBF*s in *MPL*‐mutated and *MPL*‐non‐mutated HSCs. The dot color and size indicate the average expression level (Aver. Exp) and percentage of cells expressing a given factor (Per. Exp), respectively. D) Heatmap showing significantly enriched *SREBF1*‐mediated signaling pathways by GSEA in *MPL*‐mutated compared with *MPL*‐non‐mutated ET HSCs. The pathways were collected from Reactome gene sets, WikiPathways gene sets and GO biological process. E) Bar graphs displaying relative gene expression of *SREBF1*, *SREBF2*, *FASN* in HSCs (Lin^−^CD34^+^CD38^−^CD45RA^−^CD123^−^) from NC and *MPL*‐mutated ET patients measured by RT‐qPCR (*n* = 4–5). F) Box plot depicting the expression of the Mk priming gene set^[^ ^45^ ^]^ of *MPL*‐mu HSCs and *MPL*‐non HSCs in ET patients; *P*‐values were determined by Wilcoxon rank‐sum test. G) Dot plot showing the expression of representative Mk priming genes^[^ ^45^ ^]^ across NC‐derived, *MPL*‐mutated, and *MPL*‐non‐mutated HSCs. H) Scatter plot displaying Pearson correlation between the expression of Mk priming genes and the *SREBF1* signaling genes of HSCs from *MPL*‐mutated ET patients. *P*‐values were adjusted for multiple testing using the Benjamini‐Hochberg method. I) Schematic illustration (top) of CFU‐Mk formation from UCB HSPCs (Lin^−^CD34^+^CD38^−^) with gene knockdown; bar graphs (bottom) indicating the validation of gene knockdown efficiency in UCB HSPCs (Lin^−^CD34^+^CD38^−^) using RT‐qPCR, including *SREBF1, SREBF2*, and *FASN* (*n* = 3). J) Bar graphs (left) and representative image of Mk colonies (right) illustrating a significant reduction in Mk colony formation following knockdown of *SREBF1, SREBF2, and FASN* in UCB HSPCs (Lin^−^CD34^+^CD38^−^) (*n* = 4). K) GSEA enrichment plot for the store‐operated calcium entry (SOCE) pathway (GO:0002115) in *MPL*‐mu versus *MPL*‐non HSCs. The positive normalized enrichment score (NES) indicates a coordinated increase in SOCE‐related gene expression in *MPL*‐mu HSCs. L) Dot plot showing the expression of key genes related to calcium import in *MPL*‐mutated and *MPL*‐non‐mutated HSCs. The dot color and size indicate the average expression level (Aver. Exp) and the percentage of cells expressing a given factor (Per. Exp), respectively. M) Bar graph displaying elevated gene expression of *STIM1* in HSCs (Lin^−^CD34^+^CD38^−^CD45RA^−^CD123^−^) from *MPL*‐mutated ET patients compared to NC by RT‐qPCR (*n* = 4–5). N) Scatter plot displaying Pearson correlation between the Mk priming module^[^ ^45^ ^]^ and SOCE module of HSCs from *MPL*‐mutated ET patients. *P*‐values were adjusted for multiple testing using the Benjamini‐Hochberg method. (O‐P) Relative expression of *GRP78* and *ATF4* O), and *STIM1* P) in UCB HSPCs (Lin^−^CD34^+^) after knockdown of *FASN, SREBF1*, and *SREBF2*. Expression levels were normalized to the scramble control (*n* = 4). Q) Flow cytometry analysis of calcium flux using Fluo‐4 AM in UCB HSPCs (Lin^−^CD34^+^CD38^−^) following knockdown of *FASN, SREBF1*, and *SREBF2*. Left, representative histogram showing reduced signal intensity in knockdown cells. Right, quantification of mean fluorescence intensity (MFI) normalized to scramble (*n* = 3). R) Bar graph indicating the validation of *STIM1* knockdown efficiency in UCB HSPCs (Lin^−^CD34^+^ CD38^−^) using RT‐qPCR (*n* = 3). S) Bar charts illustrating a significant reduction in CFU‐Mk formation after *STIM1* knockdown in UCB HSPCs (Lin^−^CD34^+^CD38^−^) (*n* = 4). In B, D, and K, significantly enriched pathways were determined by normal *P* <0.05 and FDR<0.25; bar color and length respectively indicate –log10 (*P* value) and normalized enrichment score (NES). In E and M, *P*‐values were calculated using an unpaired t‐test, and in I, J, O‐S, using one‐way ANOVA followed by Dunnett's multiple comparisons test. Data are shown as mean ± SEM. *P < 0.05, **P < 0.01, ***P < 0.001; ns, not significant.

To decipher the driver‐differed cellular and molecular characteristics of mutant HSCs in ET patients, we employed a modified 3′‐TARGET‐seq technique^[^ ^18^ ^]^ enabling the simultaneous capture of the targeted genotype and transcriptomic information. To ensure the genotyping fidelity, standardized samples were used as positive controls, including non‐mutated, homozygous mutated, and heterozygous mutated controls, respectively (Figures S1A and S2A, Supporting Information). After optimizing genotyping condition and ruling out the possible allele dropout, single HSCs (Lin^−^CD34^+^CD38^−^CD123^−^CD45RA^−^), obtained from untreated ET patients carrying the *JAK2* ^V617F^, *CALR*, or *MPL* mutations were subjected to 3′‐TARGET‐seq for single HSC genotype identification and unbiased transcriptomic analysis simultaneously (**Figure** **1**A; and Table S1a, Supporting Information). Additionally, to unveil the possible transcriptomic cues related to the pathogenesis of TN ET, we conducted a STRT‐seq analysis of fluorescence‐activated cell sorting (FACS)‐purified HSCs isolated from the BM of TN ET patients at diagnosis (Figure 1A). After quality control, we obtained data from ≈3000 HSCs from ET patients and ≈1100 HSCs from normal controls (NCs) (Table S1b, Supporting Information). Given the limited number of HSCs and the variable mutant allele burden across patients, our analyses were primarily based on intra‐cohort comparisons. For each mutation type, the mutated cells were compared with the non‐mutated cells from the same driver mutation cohort, as detailed in the Methods.

### 2.2 *MPL*‐Mutated HSCs in ET are Characterized by Prominent Metabolic Disturbances

To investigate the transcriptional changes potentially associated with the onset and progression of *MPL*‐mutated ET, we performed gene set enrichment analysis (GSEA) of transcriptomic data from *MPL*‐mutated and non‐mutated HSCs. The results revealed top hallmark terms (including cholesterol homeostasis, IFN response, TNFα signaling, and STAT signaling), which were significantly enriched in the *MPL*‐mutated HSCs (Figure 1B; Figure S1B,C, Supporting Information). Among them, cholesterol homeostasis was the most prominently enriched pathway (Figure 1B; Figure S1D, Supporting Information), with highly expressed key regulators of *SREBF1* ^[^ ^39^ ^]^ as well as its binding partner *SP1*,^[^ ^40^ ^]^ and *SREBF2* in *MPL*‐mutated HSCs (Figure 1C,D; Figure S1E, Supporting Information). As a result, the expression of key downstream targets of *SREBFs*, such as *FASN*, *SCD*, *FADS2*, and *ACAT2* ^[^ ^41^, ^42^, ^43^, ^44^ ^]^ was induced (Figure S1F, Supporting Information). We further confirmed the elevated expression of *SREBF1*, *SREBF2* and *FASN* by RT‐qPCR in *MPL*‐mutated ET patient HSCs (Lin^−^CD34^+^CD38^−^CD123^−^CD45RA^−^) (Figure 1E).

Next, we investigated how increased cholesterol and fatty acid metabolism might be linked to enhanced megakaryopoiesis in ET. We found the enrichment of a megakaryocytic priming gene set^[^ ^45^ ^]^ in *MPL*‐mutated HSCs (Figure 1F), including *SELP*, *RAB27B*, and *MYLK* (Figure 1G). Notably, these Mk‐priming genes were co‐expressed with *SREBF*‐regulated genes in HSCs of *MPL*‐mutated ET patients (Figure 1H). Importantly, when the expression of *SREBF1*, *SREBF2*, and *FASN* was abolished (Figure 1I; Figure S1G,H, Supporting Information), we observed diminished Mk colony formation and defective CD41a^+^CD42b^+^ generation in either umbilical cord blood (UCB) derived Lin^−^CD34^+^CD38^−^ HSPCs (Figure 1J) or Lin^−^CD34^+^ HSPCs (Figure S1I,J, Supporting Information) upon induction of Mk lineage differentiation, respectively. These findings suggest that disruption of lipid metabolism can indeed interfere with Mk lineage output.

Previous studies have shown that lipid imbalance induces lipotoxicity, disrupting endoplasmic reticulum (ER) calcium homeostasis and triggering abnormal calcium influx through the store‐operated calcium entry (SOCE) pathway;^[^ ^46^ ^]^ thereby promoting megakaryopoiesis.^[^ ^47^ ^]^ Consistently, we found that the SOCE pathway (marker gene: *STIM1* ^[^ ^47^ ^]^) was upregulated in *MPL*‐mutated HSCs (Figure 1K–M), exhibiting much higher correlation with Mk priming genes (18.2% vs 6.9%) (Figure 1N; Figure S1K, Supporting Information). Upon disruption of critical lipid metabolism regulators (*SREBF1*/*2)* and the downstream effector *FASN* in UCB‐HSPCs (both Lin^−^CD34^+^ and Lin^−^CD34^+^CD38^−^ cells), we observed reduced expression of *GRP78* and *ATF4* (key mediators of ER stress^[^ ^48^, ^49^ ^]^) (Figure 1O; Figure S1L, Supporting Information), as well as decreased *STIM1* levels (Figure 1P; Figure S1M, Supporting Information), accompanied with diminished calcium flux (Figure 1Q). To further corroborate that SOCE pathway is involved in Mk production, we reduced *STIM1* expression and revealed impaired CFU‐Mk formation and Mk differentiation (Figure 1R,S; Figure S1N,O, Supporting Information). Intriguingly, knockdown of *SREBF1*, *SREBF2*, *FASN*, and *STIM1* also hindered erythroid colony formation (BFU‐E), but had little effect on granulocyte‐macrophage colony formation (CFU‐GM) (Figure S1P, Supporting Information).

In summary, these findings support the notion that enhanced lipid metabolism, along with the aberrant regulation of the SOCE pathway in *MPL*‐mutated HSCs, may contribute to the hyper‐active megakaryopoietic potential than their non‐mutated counterparts.

### 2.3 *CALR*‐Mutated HSCs Exhibit Clear Proliferative Signatures

The molecular features of HSCs with the *CALR* mutation have not been previously examined in a highly purified population of HSCs from primary ET patients. To this end, we performed GSEA using the HALLMARK gene sets and found the *CALR*‐mutated HSCs exhibited upregulation of unfolded protein response signaling (involving, e.g., *XBP1* and *USP1*) (Figure S2B,C, Supporting Information) and enhanced cholesterol signaling (Figure S2D, Supporting Information), in accordance with prior studies.^[^ ^19^, ^28^ ^]^

**Figure 2:**
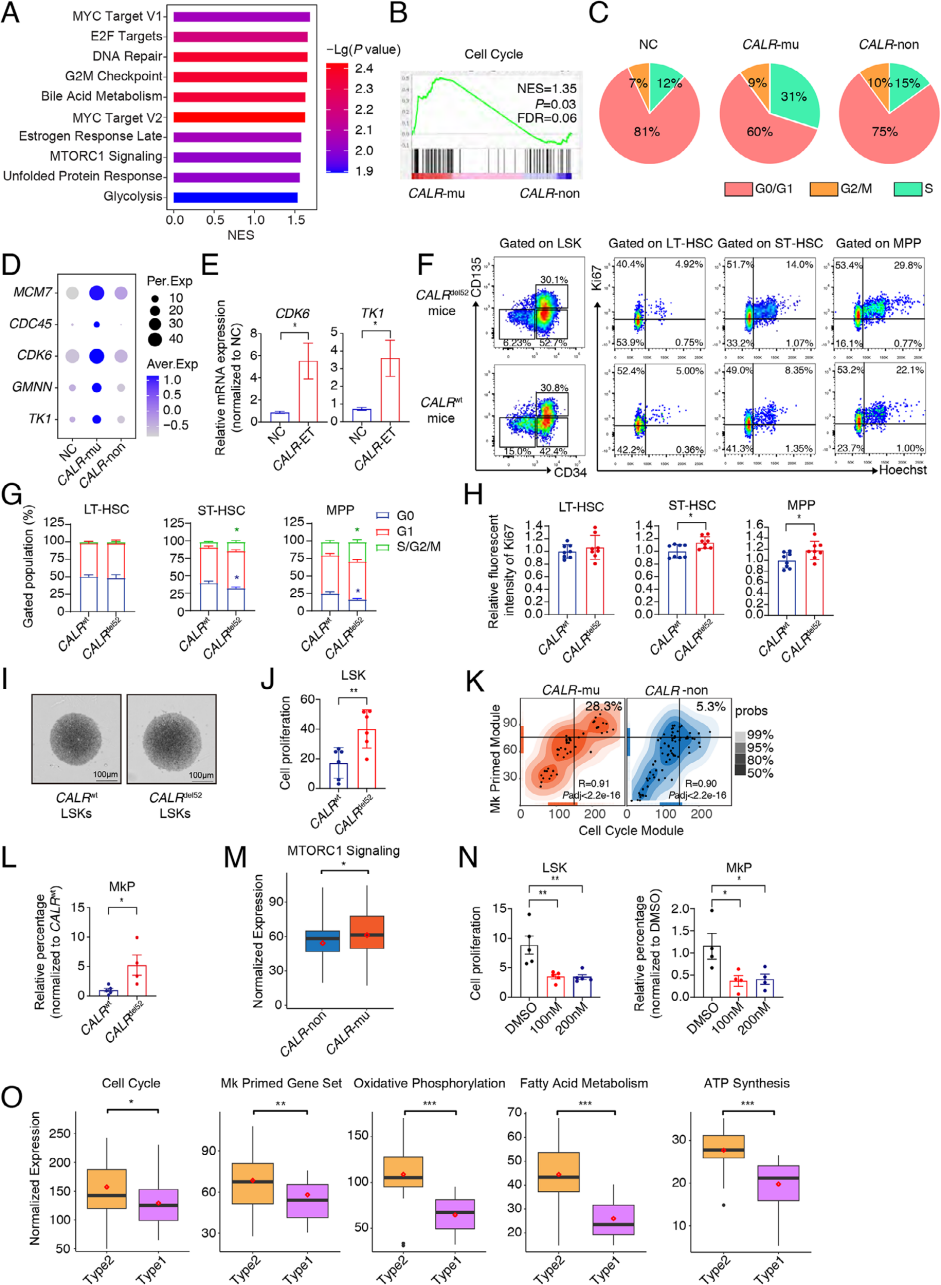
*CALR*‐mutated HSCs exhibit clear proliferative signatures. A) Bar plot showing top enriched pathways in *CALR*‐mutated (*CALR*‐mu) comparing to *CALR*‐non‐mutated ET HSCs (*CALR*‐non). B) GSEA plot showing the enrichment of WP cell cycle (WP179) in *CALR*‐mutated versus *CALR*‐non‐mutated HSCs in ET patients. C) Pie charts showing the distribution of NC, *CALR*‐mutated, and *CALR*‐non‐mutated HSCs at G0‐, G2/M‐, and S‐phase. D) The expression of key genes associated with cell proliferation in *CALR*‐mutated, and *CALR*‐non‐mutated HSCs. The dot color and size indicate the average expression level (Aver. Exp) and percentage of cells expressing a given factor (Per. Exp), respectively. E) Bar graphs displaying relative gene expression of cell cycle‐related genes, *CDK6* and *TK1*, from HSCs (Lin^−^CD34^+^CD38^−^CD45RA^−^CD123^−^) of NC and *CALR*‐ET patients by RT‐qPCR (*n* = 5‐6). F) Flow cytometry dot plots illustrating the distribution of LT‐HSC, ST‐HSC, and MPP within the LSK population (left), as well as the cell cycle distribution of each specified population (right). G) Bar plots depicting the average proportion of cells at each specified cell cycle stage between *CALR* ^del52^ and *CALR* ^wt^ mice (*n* = 7‐8). H) The mean fluorescence intensity of Ki67 in LT‐HSC, ST‐HSC, and MPP populations measured and compared between *CALR* ^del52^ and counterpart mice (*n* = 8). I) Cell growth of *CALR* ^del52^ and *CALR* ^wt^ LSKs was monitored at 72 h using light microscopy. Images were captured with an inverted microscope. J) Bar plot showing the fold expansion of LSKs illustrates the proliferative differences between *CALR* ^del52^ and *CALR* ^wt^ LSKs (*n* = 6). K) Contour plot showing the correlation between the Mk priming module^[^ ^45^ ^]^ and the Reactome cell cycle module (R‐HSA‐1640170) in *CALR*‐mutated (left) and *CALR*‐non‐mutated (right) HSCs. The horizontal and vertical lines indicate 75th percentile. Both the colored bands and marginal rugs show the distribution of each axis. L) Bar chart illustrating the increased propensity of *CALR* ^del52^ LSKs toward megakaryocytic differentiation during liquid culture assay (*n* = 4–5). M) Box plots illustrating the Hallmark mTORC1 signaling in *CALR*‐mutated and *CALR*‐non‐mutated HSCs. *P*‐values were determined by Wilcoxon rank‐sum test. N) Bar plots depicting the mTOR inhibitor rapamycin inhibits the proliferation (left) and differentiation (right) of *CALR* ^del52^ LSKs (*n* = 4–5). O) Box plots showing upregulation of Reactome cell cycle (R‐HSA‐1640170), Mk priming,^[^ ^45^ ^]^ Hallmark oxidative phosphorylation, Hallmark fatty acid metabolism, and ATP synthesis (as listed in Table S3, Supporting Information) in *CALR*‐type 2 versus *CALR*‐type 1 mutant HSCs. Wilcoxon rank‐sum test was conducted to calculate *P*‐values. In A and B, significantly enriched pathways were defined by normal *P* <0.05 and FDR<0.25; bar color and length respectively indicate –log10 (*P* value) and normalized enrichment score (NES). In E, G, H, J and L, *P*‐values were calculated using an unpaired t‐test, and in N, using one‐way ANOVA followed by Dunnett's multiple comparisons test. Data are presented as mean ± SEM. *P < 0.05, **P < 0.01, ***P < 0.001; ns, not significant.

Notably, the most prominent characteristic of *CALR*‐mutated HSCs was active cell cycle process as terms of *MYC* target gene sets, *E2F* target gene sets, and G2M checkpoint were top enriched (**Figure** **2**A–C), accompanying with the upregulation of key cell cycle mediators and checkpoints such as *CDK6*, *TK1*, and *GMNN* (Figure 2D). In concert with it, the analysis of differentially expressed genes (DEGs) in *CALR*‐mutated versus non‐mutated HSCs revealed that up to 66% of the top 50 enriched biological pathways upregulated in *CALR*‐mutated HSCs were associated with the cell cycle or DNA replication (Figure S2E and Table S2a,b, Supporting Information). We further corroborated the high expression of cell cycle‐related genes such as *CDK6* and *TK1* in *CALR*‐mutated ET patients‐derived HSCs (Lin^−^CD34^+^CD38^−^CD123^−^CD45RA^−^) (Figure 2E).

To confirm the hyperactive cell cycle characteristics on *CALR*‐mutated HSCs, we employed the *CALR* ^del52^ mouse model with an ET phenotype, which carries a human CALR C‐terminal sequence with a 52‐bp deletion in exon 9, as described in previous studies.^[^ ^50^ ^]^ First, in the LSK (Lin^−^Sca‐1^+^c‐Kit^+^) pool of *CALR* ^del52^ mice, we found a higher proportion of MPP and ST‐HSC engages in S/G2/M stage (Figure 2F,G) with enhanced Ki67 expression (Figure 2H). Second, colony assays^[^ ^51^ ^]^ showed that LSKs isolated from *CALR* ^del52^ mice formed larger colonies and had greater cell number than that of *CALR* ^wt^ counterparts (Figure 2I,J; Figure S2F, Supporting Information). Subsequently, correlation analysis revealed that genes associated with the cell cycle and Mk priming were indeed significantly co‐expressed in *CALR*‐mutated HSCs in patients (Figure 2K), consistent with the stronger Mk‐lineage bias of LSKs from *CALR* ^del52^ mice being subjected to liquid culture for 6 days in vitro (Figure 2L).

To unravel the potential underlying regulatory network of active cycle in *CALR*‐mutated HSCs, we noticed that a high expression of mTORC1 signaling (Figure 2M) as well as a variety of genes involved in metabolic processes, including mitochondrial metabolism (Figure S2G, Supporting Information), ATP synthesis (Figure S2H, Supporting Information), oxidative phosphorylation (OXPHOS), glycolysis, and fatty acid metabolism (Figure S2I, Supporting Information), in *CALR*‐mutated HSCs. Considering that mTOR signaling acts as the metabolic regulatory core,^[^ ^52^, ^53^, ^54^ ^]^ we therefore hypothesized that the heightened metabolism observed in *CALR*‐mutated HSCs (Figure S2G–I, Supporting Information) might be regulated by mTOR signaling, and the increase in energy supply may endow the mutated HSCs with a strong proliferative advantage over their unmutated counterparts. To further validate our hypothesis, rapamycin, an mTOR inhibitor, was applied. In LSKs from *CALR* ^del52^ ET mice and MEG‐01 cells, both the proliferative advantages and Mk bias were inhibited upon rapamycin treatment (Figure 2N; Figure S2J, Supporting Information). Therefore, it is likely that active mTOR signaling may partially contribute to the hyper‐proliferation as well as Mk‐lineage bias of *CALR*‐mutated HSPCs and mTOR inhibitor might be beneficial for the *CALR*‐mutated ET treatment.

Clinical studies have exposed differences in the prognosis of patients with type 1 and type 2 *CALR*‐mutated ET, whereby type 1 patients have a significantly higher risk of myelofibrotic transformation and type 2 patients have a lower risk of thrombosis, extremely high platelet counts, and an indolent clinical course.^[^ ^55^ ^]^ Nonetheless, the molecular mechanisms underlying these different disease forms remain unclear. To explore this topic, we compared the *CALR‐*mutated HSCs of type 1 and type 2 ET patients and found that type 2 *CALR*‐mutated HSCs had a stronger proliferative capacity and higher Mk priming potential, as well as increased rates of OXPHOS, fatty acid metabolism, and ATP synthesis (Figure 2O). We reasoned that the more active type 2 *CALR*‐mutated HSCs may underlie the higher platelet counts and better response to IFN treatment than the type 1 patients.^[^ ^21^ ^]^ To further validate our hypothesis, we interrogated a previously published single‐cell RNA sequencing (scRNA‐seq) dataset,^[^ ^19^ ^]^ in which *CALR*‐mutated and non‐mutated HSPCs (the total CD34^+^ BM cell population of type 1 and 2 ET patients) were differentiated by Genotyping of Transcriptomes (GoT). After dataset annotation and integrative analysis (Figure S2K–M, Supporting Information), we again observed that the *CALR*‐mutated HSPC subsets of type 2 ET patients were more active, possessing a higher Mk priming potential, as well as increased rates of cell cycling, OXPHOS, fatty acid metabolism, and ATP synthesis compared with the non‐mutated HSPCs (Figure S2N, Supporting Information). Given that IFN treatment preferentially targets activated and proliferating cells,^[^ ^20^, ^56^ ^]^ we postulated that IFN treatment may more rapidly exhaust the actively proliferating type 2 *CALR*‐mutated HSC pool and result in a better clinical response.

### 2.4 Biological Processes Shared by HSCs with Distinct Driver Mutations

**Figure 3:**
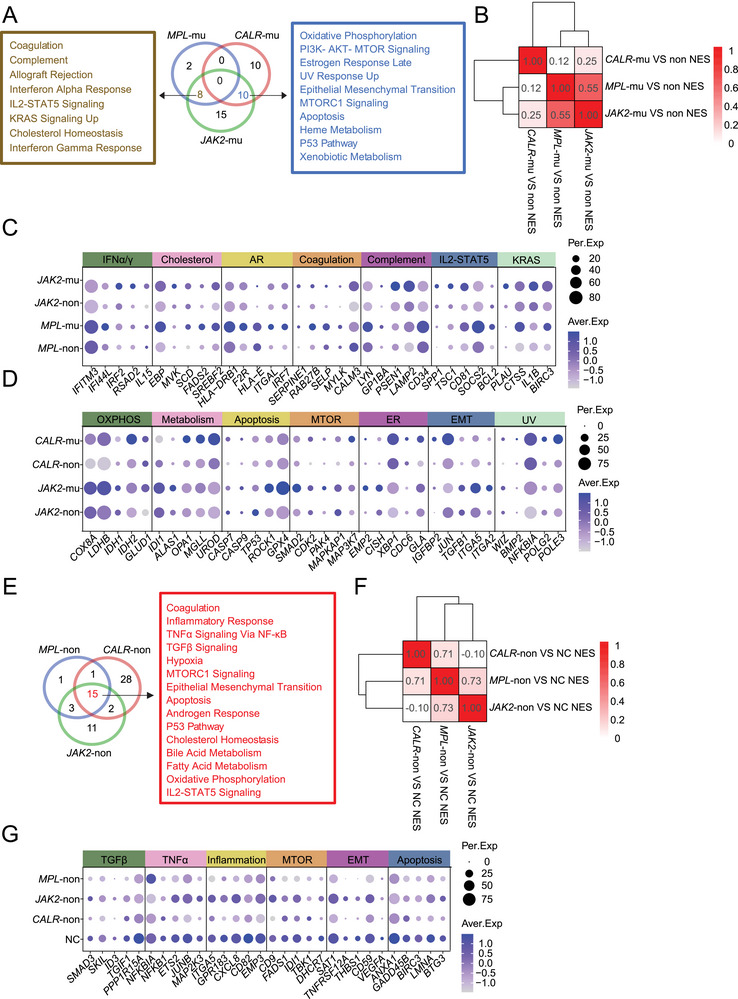
Biological processes shared by HSCs with distinct driver mutations. A) Venn diagram indicating the unique and shared enriched hallmark gene sets expressed in HSCs carrying different driver mutations versus their respective non‐mutated HSCs. Brown box: 8 gene sets were shared between *MPL‐* and *JAK2* ^V617F^‐mutated HSCs (i.e., after comparing *MPL*‐mu versus *MPL*‐non and *JAK2* ^V617F^‐mu versus *JAK2* ^V617F^‐non). Blue box: 10 gene sets were shared between *CALR‐* and *JAK2* ^V617F^‐mu HSCs. B) Heatmap showing the correlation between mutant and non‐mutated HSCs across distinct ET subtypes. Color intensity is proportional to the NES of each pairwise comparison. C) Heatmap depicting the expression of representative genes within the selected shared gene sets in *JAK2* ^V617F^‐mu, *JAK2* ^V617F^‐non, *MPL*‐mu, and *MPL*‐non HSCs. IFNα/γ, interferon‐alpha and interferon‐gamma response; AR, allograft rejection. D) Heatmap of the expression of representative genes associated within the selected shared gene sets in *JAK2* ^V617F^‐mu, *JAK2* ^V617F^‐non, *CALR*‐mu, and *CALR*‐non HSCs. OXPHOS, oxidative phosphorylation; MTOR, mTORC1 signaling; EMT, epithelial‐mesenchymal transition; ER, estrogen response late. E) Venn diagram showing the unique and shared enriched hallmark gene sets in *MPL*‐non versus NC, *CALR*‐non versus NC, and *JAK2* ^V617F^‐non versus NC HSCs. Red box: 15 enriched gene sets were shared by all ET‐non HSC subsets. F) Heatmap displaying the correlation between non‐mutated HSCs from distinct ET subtypes and NC HSCs. Color intensity is proportional to the NES for each pairwise comparison. G) Heatmap illustrating the expression of representative genes within the selected shared gene sets in NC, *JAK2* ^V617F^‐non, *MPL*‐non, and *CALR*‐non HSCs. TGFβ, TGF‐beta signaling; TNFα, TNF‐alpha signaling via NF‐κB; MTOR, mTORC1 signaling; EMT, epithelial‐mesenchymal transition.

Although the driver mutations targeting *MPL*, *CALR*, and *JAK2* ^V617F^ in ET all induce excessive thrombocytosis, they are associated with different disease outcomes and prognosis. Therefore, we next sought to investigate the shared and unique molecular properties arising from different driver mutations in HSCs. To this end, we began to interrogate our previously published dataset generated from *JAK2* ^V617F^‐mutated HSCs.^[^ ^16^ ^]^ We re‐assessed the GSEA profiles of these cells by comparing mutated and non‐mutated HSCs in ET patients. Our results revealed an enrichment of terms such as inflammation response, IFN signaling, and cholesterol homeostasis in *JAK2* ^V617F^ ‐mutated versus non‐mutated HSCs (Figure S3A, Supporting Information). Next, we extracted and integrated all the enriched GSEA hallmark terms from the comparison of *MPL*‐mutated versus *MPL*‐non‐mutated HSCs, *JAK2* ^V617F^ ‐mutated versus *JAK2* ^V617F^ ‐non‐mutated HSCs, and *CALR*‐mutated versus *CALR*‐non‐mutated HSCs (**Figure** **3**A; Figure S3B, Supporting Information). Correlation analysis based on these enriched terms showed that *MPL*‐ and *JAK2* ^V617F^‐mutated HSCs had similar properties, while those of *CALR*‐mutated HSCs were more unique (Figure 3A,B). Detailed analysis of these enriched terms showed that genes implicated in eight common signaling pathways or biological processes, including Mk‐ and platelet‐associated coagulation (e.g., *RAB27B*, *SELP*), inflammatory and IFN response pathways (e.g., *IFITM3*, *IRF2*), KRAS pathways (e.g., *IL1B, BIRC3*), cholesterol hemostasis (e.g., *SCD*, *FADS2*) and allograft rejection pathways (e.g., *HLA‐E, ITGAL*), were shared between *MPL*‐ and *JAK2* ^V617F^ ‐mutated HSCs (Figure 3A,C). Meanwhile, we also identified 10 signaling pathways or biological processes, which were shared between *CALR*‐ and *JAK2* ^V617F^‐mutated HSCs; these signaling pathways mainly belong to active metabolic processes, such as OXPHOS (e.g., *LDHB*, *COX8A*), heme metabolism (e.g., *ALAS1*, *UROD*), fatty acid metabolism (e.g., *IDI1*, *OPA1*), and even xenobiotic metabolism (e.g., *MGLL*) (Figure 3A,D). Unexpectedly, no common signaling pathways or biological processes were shared across all three types of mutated HSCs, probably due to the limited number of hallmark gene sets included in the database (Figure 3A).

In addition to expounding characteristics of mutant cells, we found that non‐mutated HSCs of ET patients also differed from NC HSCs. Moreover, correlation analysis showed that the non‐mutated HSCs across the ET spectrum had relatively similar correlation scores (Figure 3F). Comparison with NC HSCs revealed 15 shared biological processes among the three types of non‐mutated HSCs of ET patients, which accounted for 75% of the enriched terms in NC versus *MPL*‐non‐mutated, 32.6% of the enriched terms in NC versus *CALR*‐non‐mutated, and 48.4% of the enriched terms in NC versus *JAK2* ^V617F^ ‐non‐mutated HSCs (Figure 3E). This result implies that, regardless of the type of driver mutation, the BM microenvironment may be similarly affected in each type of ET patient. For example, TGFβ signaling, TNFα signaling, and EMT were significantly downregulated in the non‐mutated HSCs of all the ET subtypes evaluated (Figure 3G; Figure S3C, Supporting Information). TGFβ signaling controls the quiescence and self‐renewal of HSCs, and thus, potentially contributes to HSC aging. Reduced TGFβ signaling may decrease HSC self‐renewal in favor of the myeloid‐oriented differentiation of HSCs.^[^ ^57^ ^]^ Its downregulation in non‐mutated HSCs might therefore lower the threshold of HSC activation and drive HSC proliferation and differentiation. In line with this hypothesis, we found that the expression of *MYC* targets genes was significantly elevated in non‐mutated HSCs (Figure S3C, Supporting Information, right panel). Besides, the unique molecular features of non‐mutated HSCs within each of the three types of ET were also analyzed (Figure 3E; Figure S3D, Supporting Information).

Taken together, these findings indicate that the common features of non‐mutated HSCs within each of the three types of ET may partially reflect the influence of the abnormal BM microenvironment in MPNs. The dysregulation of multiple signaling pathways may alter the state of non‐mutated HSCs (e.g., leaving quiescence to active state) or impair their survival, which ultimately might promote the pathogenesis of MPN.

### 2.5 Identification of a Stem Cell Subset Exhibiting Malignant Features in TN ET HSCs

**Figure 4:**
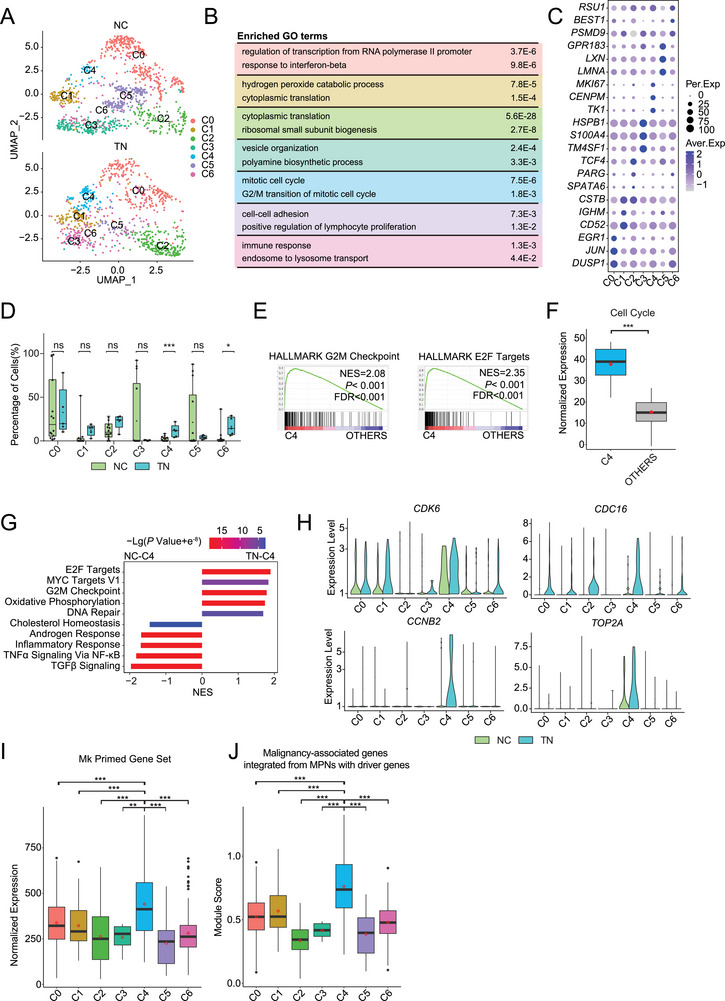
Identification of a stem cell subset exhibiting malignant features in TN ET HSCs. A) Uniform manifold approximation and projection (UMAP) plot displaying the clusters of TN and NC HSCs after CCA integration. B) Enriched GO terms associated with the signature genes of each cluster. Adjusted *P*‐values are shown. C) Dot plot depicting the expression of signature genes in each cluster. The dot color and size indicate the average expression level (Aver. Exp) and percentage of cells expressing a given factor (Per. Exp), respectively. D) Column plot showing the frequency of each cluster in NC (green) and TN (blue) HSC populations. E) GSEA plot displaying the enrichment of Hallmark G2/M checkpoint (left) and Hallmark E2F targets (right) in C4 versus the other HSCs isolated from TN ET patients. F) Box plot comparing the expression of the Kegg cell cycle pathway (hsa04110) in C4 and the other HSCs isolated from TN ET patients, both median (black bar) and mean (red diamond) expression levels are shown. G) Bar graph illustrating the enrichment of top 5 hallmark terms in the C4 subcluster of NCs and TN ET patients. H) The expression of key cell cycle mediators (e.g., *CDK6, CDC16, CCNB2*, and *TOP2A)* in each HSC cluster in NCs (green) and TN ET patients (blue). I) Box plot showing that C4 expressed the highest level of genes associated with Mk priming among all the HSC clusters. J) Box plot illustrating the mutated‐HSC signature score of each identified cluster in TN HSCs. Mutant features were defined as a combination of genes highly expressed in mutant HSCs harboring distinct driver mutations, compared with their respective non‐mutated counterparts. In E and G, significantly enriched pathways were defined by normal *P* <0.05 and FDR<0.25; bar color and length respectively indicate –log10 (*P* value) and normalized enrichment score (NES). In D and F, *P*‐values were calculated using Wilcoxon rank‐sum test, and in I and J, using Wilcoxon rank‐sum test followed by Dunn's multiple comparisons test. *P < 0.05, **P < 0.01, ***P < 0.001; ns, not significant.

To date, no driver mutation has been identified in TN ET patients. To better understand the pathogenesis of TN ET, we conducted scRNA‐seq using highly purified HSCs. Specifically, we sought to analyze the heterogeneity of HSCs, as a certain heterogeneous HSC cluster has been linked to the pathogenesis of ET.^[^ ^1^, ^16^, ^58^ ^]^ We then performed dimensionality reduction using the uniform manifold approximation and projection (UMAP) method and clustered TN HSCs into seven clusters (**Figure** **4**A). Based on the marker genes of each cluster (Figure 4B,C), C0 was related to transcription regulation and IFN response (e.g., *DUSP1* and *JUN*); C1 was correlated with metabolic processes such as oxidation‐reduction and translation process (e.g., *CD52, IGHM*); C2 was related to ribosomal biogenesis (e.g., *SPATA6, PARG*); C3 was associated with polyamine biosynthesis and vesicle organization (e.g., *TM4SF1, HSPB1*); C4 was characterized by active cell cycling (e.g., *TK1*, *CENPM)*, C5 was characterized by cell adhesion and lymphocyte proliferation (e.g., *GPR183*, *LMNA*); and C6 was associated with the immune response and lysosome transport (e.g., *BEST1*, *RSU1*) (Figure 4B,C; Figure S4A,B, Supporting Information).

We noted that patients with TN ET had significantly larger C4 and C6 populations than NCs (Figure 4D). Consistently, the GSEA of C4 cells from TN ET patients and NCs also confirmed the greater proliferative potential of the TN C4 cells (Figure 4E,F). For example, cell‐cycle regulators (e.g., *CDK6*, *CDC16*) and mediators (e.g., *CCNB2*) were expressed at much higher levels in TN than in NC C4 cells (Figure 4G,H). Moreover, because C4 cells expressed markers of highly active cell proliferation (Figure 4B,C,E–H), they likely had the strongest proliferative capacity among all the subsets of NC and TN HSCs. Apart from proliferation, C4 TN cells were highly enriched in metabolic processes such as OXPHOS (Figure 4G). As Mk priming is an important pathogenic transcriptomic signature, we also noted that the C4 subpopulation exhibited the most striking Mk‐priming signature among the different TN HSC clusters (Figure 4I). While TN C6 cells were associated with a higher IFN response than their NC equivalents (Figure S4D, Supporting Information), they had a reduced inflammatory response signature (Figure S4C, Supporting Information).

To assess the malignant potential of each TN subpopulation, we scored the expression of signature genes derived from the *JAK2* ^V617F^ */CALR/MPL*‐mutated HSCs in distinct clusters of TN HSCs. We found that the C4 subpopulation exhibited the highest expression of transcriptomic signatures resembling those of mutant cells (Figure 4J). However, whether this malignant‐like HSC subset underlies the pathogenesis of TN ET or arises in response to the inflammatory stimuli associated with ET remains unclear and warrants further investigation.

### 2.6 The C5 Cluster is Largely Absent from All ET Subtypes

**Figure 5:**
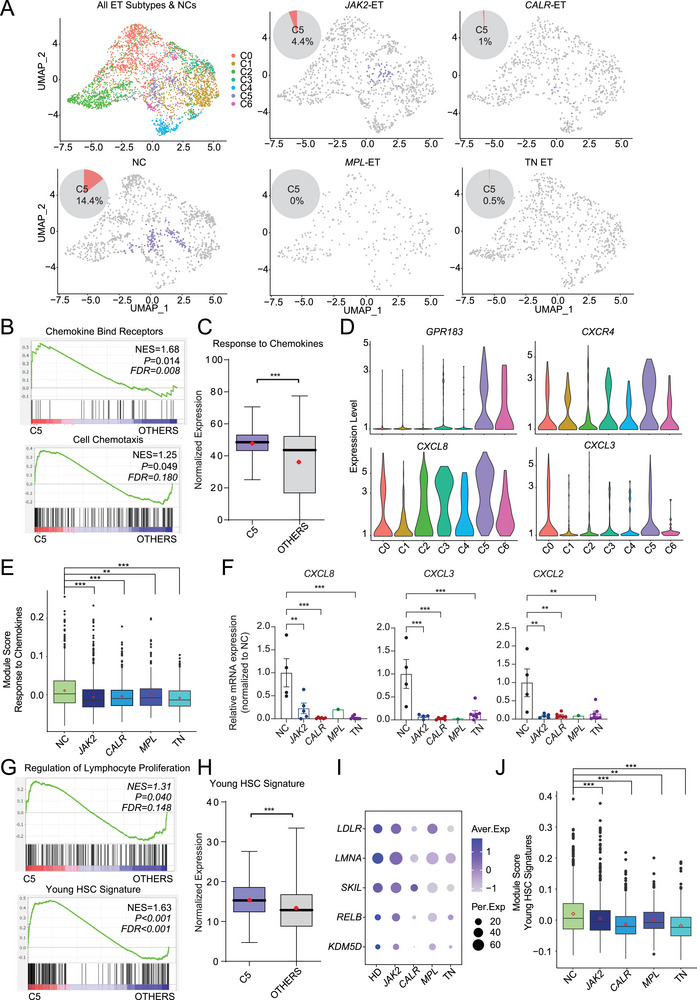
The C5 cluster is largely absent from all ET subtypes. A) UMAP plot showing the clustering of all HSCs from NCs and *JAK2* ^V617F^‐, *CALR*‐, and *MPL‐*mutated, and TN ET patients. HSCs were clustered into seven clusters. The C5 cluster is highlighted in purple. The pink slice in the pie chart indicates the frequency of the C5 subset in different sample types. B) GSEA plots demonstrating the enrichment of genes associated with chemokine receptors (R‐HSA‐380108) (top) and GOBP cell chemotaxis (GO:0060326) (bottom) in C5 versus other HSC subsets. C) Box plot showing the expression of GOBP response to chemokines (GO:1990868) in C5 versus other clusters; both median (black bar) and mean (red diamond) expression levels are shown. D) Violin plots depicting that *GPR183, CXCR4*, *CXCL8*, and *CXCL3* were significantly enriched predominantly in C5. E) Box diagram describing the gene module scores (calculated using the Seurat R package) in GOBP response to chemokines (GO:1990868) in all HSCs of NCs and ET patients. F) Quantitative real‐time PCR (RT‐qPCR) was used to measure the relative expression of *CXCL2*, *CXCL3*, and *CXCL8* in FACS‐purified HSCs (Lin^−^CD34^+^CD38^−^CD123^−^CD45RA^−^) from the bone marrow (BM) of NCs and ET patients (*n* = 1–7). Each dot indicates a single individual. Data are presented as the mean ± SEM. P‐values were determined by one‐way ANOVA followed by Dunnett's multiple comparisons test. *P < 0.05, **P < 0.01, ***P < 0.001. G) GSEA plot depicting the expression of GO regulation of lymphocyte proliferation pathway (GO:0050670)(top) and young HSC signature genes^[^ ^59^ ^]^ (Table S3, Supporting Information) (bottom) in C5 versus other HSC subsets. H) Box plot showing the expression of young HSC signature genes in C5 and other HSC clusters. I) Dot plot displaying the expression of genes associated with a young HSC signature in ET patients and NCs. The dot color and size indicate the average expression level (Aver. Exp) and the percentage of cells expressing a given factor (Per. Exp), respectively. J) Box plot illustrating the expression scores of HSCs with a young signature in NCs and ET patients. In B and G, significantly enriched pathways were determined by normal *P* <0.05 and FDR<0.25. In C and H, *P*‐values were calculated using Wilcoxon rank‐sum test; in E and J, using Wilcoxon rank‐sum test followed by Dunn's multiple comparisons test. *P < 0.05, **P < 0.01, ***P < 0.001; ns, not significant.

To determine whether there are features shared across all ET subtypes, we integrated all ET HSC data and clustered them by UMAP (**Figure** **5**A; Figure S5A–C, Supporting Information). After comparing the frequency of each cluster, we discovered that the proportions of C3 and C5 were significantly lower in all examined ET subtypes than in NCs (Figure S5C, Supporting Information). Particularly, C5 was nearly completely absent from the HSC repertoire of *CALR*‐mutated, *MPL*‐mutated, and TN patients (Figure 5A).

Molecular characterization of C5 revealed that it was significantly and uniquely enriched for genes associated with chemokine production and chemotaxis (e.g., *CXCL8* and *GPR183*) (Figure 5B–D; Figure S5B, Supporting Information). This result implies that chemotaxis signaling genes were largely not expressed in ET HSCs. In accordance, the chemokine response pathway was significantly downregulated in all ET subtypes (Figure 5E). Moreover, RT‐qPCR analysis confirmed that the expression of key chemokine‐associated genes decreased in purified BM HSCs (Lin^−^CD34^+^CD38^−^CD123^−^CD45RA^−^) from different subtypes of ET patients versus those from NCs (Figure 5F).

Next, we aimed to decipher the differentiation potential and underlying function of C5 in ET. First, trajectory analysis has revealed that C5 HSC subset exhibited strongest lymphoid bias with a pronounced partial preference toward lymphoid‐primed multipotent progenitors (LMPPs), in line with GSEA results (Figure 5G; Figure S5F, Supporting Information), while the remaining other subsets showed more myeloid bias (Figure S5D,E, Supporting Information). Aligning with the relative strong lymphoid lineage potential, gene sets related to young HSCs^[^ ^59^ ^]^ and HSC homing capacity^[^ ^60^ ^]^ were indeed significantly enriched in the C5 cluster (Figure 5G,H; Figure S5B, Supporting Information). As the consequence of the absence of C5, the expression of gene set related with young HSCs, such as *LMNA* and *RELB*,^[^ ^61^, ^62^ ^]^ was downregulated in all ET subtypes (Figure 5I,J). These findings imply that the absence of C5 subset from HSC pool in MPN patients would possibly skew into myeloid‐biased differentiation with compromised lymphoid lineage potential and reduced self‐renewal capacity.

### 2.7 HSCs Lacking CXCR4^+^ Subset Exhibit Pronounced Myeloid Lineage Bias

**Figure 6:**
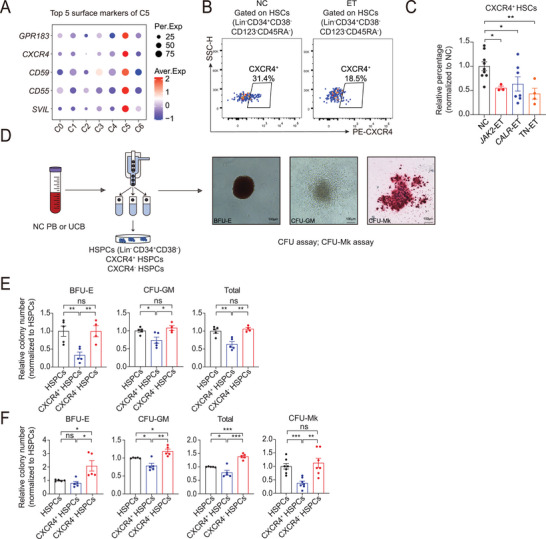
HSCs lacking CXCR4^+^ subset exhibit pronounced myeloid lineage bias. A) A heatmap dot plot showing the expression of top 5 surface marker genes in C5. The red and blue colors indicate the row‐scaled expression of the indicated genes. B) Flow cytometry dot plots showing the gating strategy used for the analysis and isolation of CXCR4^+^ HSCs (Lin^−^CD34^+^CD38^−^CD123^−^CD45RA^−^). C) Bar graph showing the relative percentage of CXCR4^+^ HSCs (Lin^−^CD34^+^CD38^−^CD123^−^CD45RA^−^) in NCs and ET patients. Each dot represents a single individual. (*n* = 3–9). D) Experimental scheme for the colony‐forming unit (CFU) assay. Total, CXCR4^+^, or CXCR4^−^ HSPCs (Lin^−^CD34^+^CD38^−^) were FACS‐sorted from umbilical cord blood (UCB) or peripheral blood (PB) of NCs and then subjected to CFU assays. Then BFU‐E, CFU‐GM, and Mk colonies were identified and counted after 14 days of culture. The images (right) show the representative BFU‐E, CFU‐GM, and Mk colonies. E) Bar graphs showing the relative numbers of BFU‐E, CFU‐GM, and total myeloid colonies from total, CXCR4^+^, and CXCR4^−^ HSPCs (Lin^−^CD34^+^CD38^−^) isolated from human PB (*n* = 4‐5). F) Bar graphs displaying the relative numbers of BFU‐E, CFU‐GM, total myeloid, and Mk colonies from total, CXCR4^+^, and CXCR4^−^ HSPCs (Lin^−^CD34^+^CD38^−^) isolated from human UCB (*n* = 5–7). Data are presented as the mean ± SEM. In C, *P*‐values were calculated using one‐way ANOVA followed by Dunnett's multiple comparisons test, in E‐F, using one‐way ANOVA followed by Tukey's multiple comparisons test. *P < 0.05, **P < 0.01, ***P < 0.001; ns, not significant.

To further investigate the functional role of the C5 subset at the experimental level, we first evaluated the surface marker expression aiming to either deplete or isolate this subset from the normal HSC compartment for use in functional assays. Two genes, *GPR183* and *CXCR4*, caught our attention (**Figure** **6**A). A prior study showed that *GPR183* expression is strongly correlated with high lymphoid lineage potential,^[^ ^63^ ^]^ while *CXCR4* is associated with the homing, quiescence, and lymphopoiesis of HSCs.^[^ ^60^, ^64^, ^65^, ^66^, ^67^ ^]^ The population of GPR183 expressed in HSCs is very small (<10%), we then assessed the fraction of CXCR4^+^ HSCs (Lin^−^CD34^+^CD38^−^CD123^−^CD45RA^−^) in the BM of ET patients and NCs by flow cytometry. We found that their frequency was indeed significantly reduced in *JAK2* ^V617F^‐mutated, *CALR‐*mutated, and TN ET patients (Figure 6B,C). Next, to explore the differentiation preference of CXCR4^+^ HSCs subset and the influence of a reduction in CXCR4^+^ HSC subpopulation on hematopoietic lineage differentiation, we performed colony‐forming unit (CFU) assays using FACS‐sorted total HSPCs (Lin^−^CD34^+^CD38^−^), CXCR4^+^, and CXCR4^−^ HSPCs isolated from the UCB or peripheral blood (PB) of NCs (Figure 6D). We found that CXCR4^−^ HSPCs (Lin^−^CD34^+^CD38^−^) generated more myeloid and erythroid colonies than CXCR4^+^ HSPCs (Figure 6E,F). Furthermore, as ET is characterized by sustained megakaryopoiesis and a persistently elevated platelet production, we conducted CFU‐Mk assays. The CXCR4^−^ HSPCs (Lin^−^CD34^+^CD38^−^) yielded more Mk colonies than the CXCR4^+^ ones (Figure 6F); in accordance, PB‐derived CXCR4^−^ HSPCs (Lin^−^CD34^+^) produced more Mk colonies than the CXCR4^+^ ones (Figure S6A,B, Supporting Information). In summary, these data suggest that the loss of C5 skews HSPCs toward myeloid lineage differentiation, resulting in increased Mk generation.

### 2.8 CXCR4^+^ HSCs Transplantation Delays MPN Onset

Next, we sought to validate the role of the C5 subset in vivo. We first examined whether the characteristics and phenotypic features of the C5 subset observed in mice were consistent with those found in patients.

**Figure 7:**
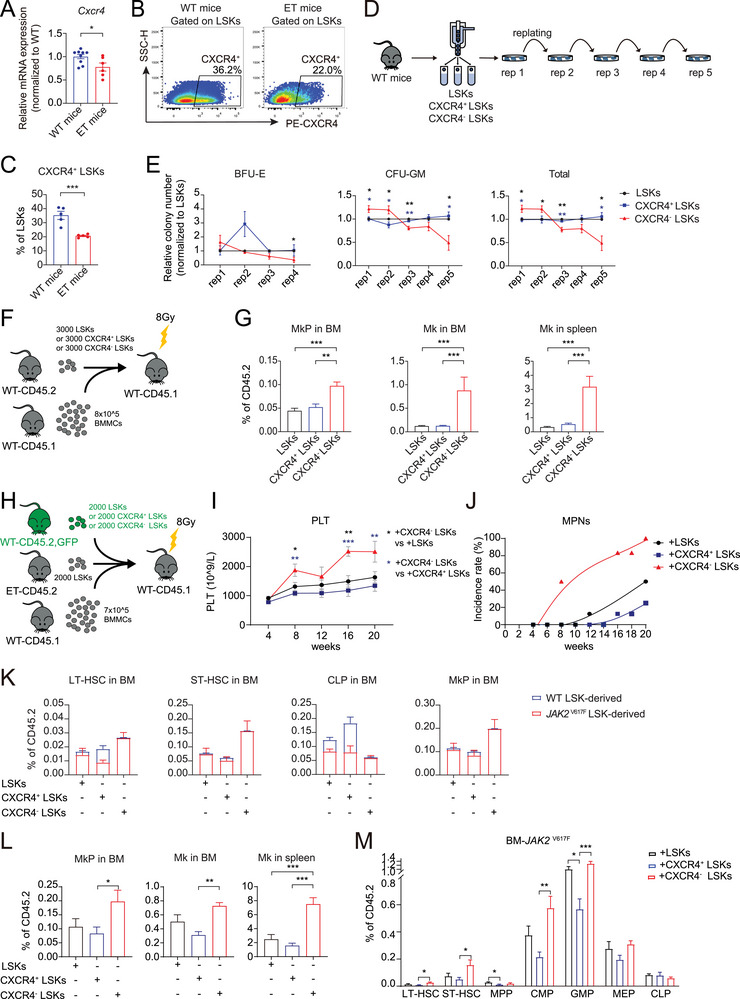
CXCR4^+^ HSCs transplantation delays MPN onset. A) Bar graph showing the relative expression of *Cxcr4* in the BM LSKs (Lin^−^Sca‐1^+^c‐Kit^+^ cells) of wildtype (WT) and *JAK2* ^V617F^ ET mice. Each dot indicates a single mouse (*n* = 6–9). B) Representative flow cytometry plot of CXCR4^+^ LSKs in WT and *JAK2* ^V617F^ ET mice. C) The percentages of CXCR4^+^ LSKs in WT and *JAK2* ^V617F^ ET mice. Each dot indicates a single mouse (*n* = 5). D) Schematic of the replating assays for LSKs. In brief, FACS‐sorted LSKs, CXCR4^+^ LSKs, and CXCR4^−^ LSKs were subjected to CFU assays. After 12 – 14 days of culture, the BFU‐E and CFU‐GM colonies were counted, then the cells were collected, washed and subjected to another CFU assay. Five clonogenic assays were executed in total. E) Line graphs displaying the relative numbers of BFU‐E, CFU‐GM, and total myeloid colonies formed during the serial replating assay (*n* = 5). F) Schematic diagram of the intra‐BM competitive transplantation procedure. Briefly, 8 × 10^5^ control donor BM cells (CD45.1) were transplanted together with 3000 competitor LSKs (CD45.2) into lethally irradiated WT recipient mice (CD45.1). The recipient mice were then sacrificed and their BM and spleen cells were harvested and analyzed by flow cytometry. G) The frequency of competitor‐derived MKP and Mk in the BM, as well as Mk in the spleen of recipient mice transplanted with total, CXCR4^+^, and CXCR4^−^ LSKs at 24 weeks post‐transplantation (*n* = 5‐11). H) Schematic diagram of the intra‐BM competitive transplantation procedure. 7 × 10^5^ control donor BM cells (CD45.1) and 2000 *JAK2* ^V617F^‐LSKs(CD45.2) were transplanted together with 2000 competitor GFP‐WT‐LSKs (CD45.2, CXCR4^+^ LSKs, CXCR4^−^ LSKs and total LSKs respectively) into lethally irradiated WT recipient mice (CD45.1). I) Line graph depicting the peripheral blood platelet counts of recipient mice, measured at four‐week intervals (*n* = 6–9). J) The curve illustrating the incidence rate of MPNs in recipient mice over time. K) Bar plots displaying the frequency of *JAK2* ^V617F^ LSK‐ and WT LSK‐derived LT‐HSC, ST‐HSC, CLP, and MkP in the BM (CD45.2) of recipient mice transplanted with total, CXCR4^+^, and CXCR4^−^ LSKs (*n* = 6–9). L) Bar charts depicting the decreased differentiation potential of *JAK2* ^V617F^‐ LSKs toward the megakaryocytic lineage in both BM and spleen cells (CD45.2) following supplementation with CXCR4^+^ LSKs (*n* = 6–9). M) Bar chart illustrating the stem and progenitor cell characteristics of BM cells derived from *JAK2* ^V617F^‐ LSKs, highlighting the reduced myeloid lineage differentiation potential following supplementation with CXCR4^+^ LSKs (*n* = 6–9). Data are presented as mean ± SEM. In A and C, *P*‐values were calculated using an unpaired t‐test, and in E, G, I, L, and M, by one‐way ANOVA with Tukey's test. *P < 0.05, **P < 0.01, ***P < 0.001; ns, not significant.

It was observed that the expression of *Cxcr4* was decreased in the LSKs of *JAK2* ^V617F^ transgenic mice by RT‐qPCR (**Figure** **7**A; and Table S1C, Supporting Information). Then, flow cytometric analysis showed that the proportion of CXCR4⁺ LSKs was reduced in *JAK2* ^V617F^ ET mice than in WT controls (Figure 7B,C). Moreover, we FACS‐sorted LSKs, CXCR4^+^ LSKs, and CXCR4^−^ LSKs from WT mice and conducted colony forming and serial replating assays (Figure 7D). CXCR4^−^ LSKs showed stronger myeloid and erythroid colony‐forming ability than CXCR4^+^ LSKs (Figure 7E; Figure S7A, Supporting Information), in agreement with CFU results performed with PB‐ or UCB‐derived HSCs. Additionally, the colony‐forming ability of CXCR4^–^ LSKs decreased at a higher rate compared to CXCR4^+^ LSKs as the number of replating cycles increased (Figure 7E), indicating that the absence of CXCR4^+^ subpopulation in the LSK pool may impair their long‐term repopulation and self‐renewal capacity.

Next, to fully address the hematopoietic reconstitution and lineage differentiation potential of LSKs with or without C5, we performed a competitive transplantation assay using C57BL/6‐CD45.1 competitor BM‐derived mononuclear cells (BMNCs), and FACS‐sorted C57BL/6‐CD45.2 donor total LSKs, CXCR4^+^ LSKs, or CXCR4^−^ LSKs (Figure 7F). Given that CXCR4 is essential for HSC homing, we performed intra‐BM transplantation; however, we still observed significantly lower rates of PB reconstitution by CXCR4^−^ LSKs than by total LSKs and CXCR4^+^ LSKs at 24 weeks post‐transplantation (Figure S7B,C, Supporting Information). It has been demonstrated that CXCR4 is not only crucial for HSC homing but also essential for HSC retention in their niche.^[^ ^68^ ^]^ Thus, the reduced reconstitution rate of CXCR4^−^ LSKs suggested impaired homing to, or retention within, the niche, further supporting their diminished reconstruction capability compared to CXCR4^+^ LSKs.

In spite of the lower reconstitution ability of CXCR4^−^ LSKs in recipients, lineage analysis revealed that the frequencies of Lin^−^Sca‐1^+^c‐Kit^+^CD34^+^CD135^−^ (ST‐HSC), Lin^−^Sca‐1^−^c‐Kit^+^CD34^+^CD16/32^lo^ (CMP), and Lin^−^Sca‐1^−^c‐Kit^+^CD34^+^CD16/32^hi^ (GMP) cells in the CXCR4^−^ LSK group were significantly higher than that in the CXCR4^+^ LSK group (Figure S7D,E, Supporting Information), suggesting that HSPCs lacking CXCR4 exhibited higher myeloid lineage potential. Moreover, the proportions of Lin^−^Sca‐1^−^c‐Kit^+^CD150^+^CD41^+^ (MkP), CD41^+^CD42d^+^ (Mk) within the BM and spleen CD45.2^+^ cell populations of CXCR4^−^ LSK group were higher than those of the CXCR4^+^ LSK group (Figure 7G). This result further implied that CXCR4^−^ LSKs, namely LSKs with C5 loss, possess stronger myeloid (particularly Mk) differentiation potential yet impaired graft capability.

To understand the effects of C5 subset on MPN initiation, we conducted a competitive bone marrow transplantation experiment (Figure 7H). CD45.2^+^ GFP‐labeled CXCR4^+^/CXCR4^−^/total LSK cell populations, along with cells derived from ET mice carrying the *JAK2* ^V617F^ mutation and CD45.1^+^ WT BMNCs for protection, were transplanted into CD45.1 wild‐type mice. Peripheral blood parameter in these mice was subsequently detected to monitor the development of MPN. Mice transplanted with CXCR4^+^ LSKs exhibited significantly lower platelet (PLT) counts (Figure 7I; Figure S7F, Supporting Information) and a notable delay in MPN onset (Figure 7J). At 20 weeks post‐transplantation, biopsies of bone marrow from the CXCR4^+^ LSKs‐supplemented group displayed the lowest Mk levels among all groups (Figure S7G, Supporting Information). Furthermore, markedly reduced proportion of MkP and Mk within the bone marrow and spleen also hinted the inhibited megakaryopoiesis in CXCR4^+^ LSKs‐supplemented group, when compared with CXCR4^−^ LSKs group (Figure 7L; Figure S7H, Supporting Information).

Additionally, the clonal proliferation of *JAK2* ^V617F^‐mutated cells in the bone marrow of these mice was markedly inhibited by supplementing CXCR4^+^ LSKs (Figure 7K–M; Figure S7I, Supporting Information). Specifically, there was a significant reduction in MkP and Mk derived from *JAK2* ^V617F^‐mutated LSKs compared to that from CXCR4^−^ LSKs (Figure 7K,L), along with significantly lower percentage of LT‐HSC, ST‐HSC, CMP, and GMP originating from *JAK2* ^V617F^‐mutated LSKs in the CXCR4^+^ LSKs‐supplemented mice (Figure 7M).

Therefore, it is likely that the loss of the C5 subset is not merely a concomitant feature of ET pathogenesis since replenishing the missing C5 subset can inhibit the clonal proliferation of MPN‐mutated cells and lead to an alleviation of MPN onset and MPN phenotype.

## 3 Discussion

HSCs are heterogeneous in their self‐renewal capacity and intrinsic lineage bias;^[^ ^69^ ^]^ however, the enigma of genetically distinct HSC clones harboring *JAK2* ^V617F^, *CALR*, or *MPL* mutations but giving rise to similar clinical features remains unclear, such as in ET. One of the key questions in the field is whether *JAK2* ^V617F^‐, *MPL‐*, and *CALR*‐mutated HSCs exhibit distinct molecular signatures that not only differentiate them from their respective non‐mutated counterparts but also from one another. Defining such distinct characteristics could aid the development of therapies that target HSC clones with a specific driver mutation, while sparing normal hematopoiesis. Here, we used single‐cell transcriptomic sequencing, coupled with single‐cell driver mutation detection, to specifically distinguish mutated HSCs from their non‐mutated counterparts within the same patient with ET. It revealed that *MPL*‐mutated HSCs are characterized by hyperactive cholesterol and lipid metabolism, which likely contributes to the enhanced megakaryopoiesis. These findings suggest that targeting aberrant lipid metabolic processes may offer another promising therapeutic approach for *MPL*‐mutated ET. *CALR*‐mutated HSPCs are characterized by active cell cycling and an elevated unfolded protein response.^[^ ^19^, ^28^, ^70^ ^]^ Here, we further highlighted that the highly active proliferative properties are evident at the stem cell level, suggesting that *CALR* mutations promote hyperactive HSC behavior. Moreover, comparisons of *JAK2* ^V617F^ *‐* and *MPL‐*mutated HSCs revealed that such properties were enriched most in *CALR*‐mutated cells, suggesting that perturbing cell cycle might potentially benefit ET patients carrying *CALR* mutations more in addition to the standard therapies.

Beyond mutated cells, non‐mutated HSCs also reside within the BM microenvironment of MPN patients, whose molecular alterations may partially reflect microenvironment changes. We demonstrated that the non‐mutated HSCs of MPN patients differed from those of NCs, consistent with prior studies.^[^ ^18^, ^19^ ^]^ Interestingly, non‐mutated HSCs exhibited remarkable transcriptional similarity across different MPN subtypes, irrespective of the driver mutation. This observation suggests that, although mutant HSCs are genetically distinct, they may induce shared microenvironmental alterations that, in turn, influence non‐mutated HSCs.

To date, extensive efforts to identify potential driver mutations in TN ET have failed to reveal recurrent clonal mutations in a substantial proportion of patients.^[^ ^71^, ^72^ ^]^ Our findings point to an alternative layer of disease regulation, suggesting that transcriptional reprogramming may contribute to the pathogenesis of TN ET. Our data unveiled an HSC subset (C4) in TN ET, exhibiting features similar to *JAK2* ^V617F^ *‐*, *CALR‐*, or *MPL*‐mutated HSCs, including enhanced cell cycling and megakaryocytic priming, suggesting a possibility that this subset might contribute transcriptionally to the pathogenesis of TN ET. Yet, we cannot exclude the alternative explanation that such malignant‐like reprogramming reflects an adaptive response to the inflammatory microenvironment characteristic of ET. Distinguishing between these possibilities will require future studies integrating longitudinal data and functional validation.

Another key finding of this study is the consistent depletion of a specific HSC subset (C5) across all MPN subtypes. Compared with other clusters, C5 showed elevated expression of genes involved in chemotaxis and lymphopoiesis. Depletion of C5 cells from the HSC or HSPC compartments by surface marker CXCR4 enhanced the colony‐forming capacity of megakaryocytic, erythroid, and myeloid lineages, but impaired long‐term repopulating potential. This finding aligns with the hypothesis that altered HSC pool composition may contribute to disease onset.

Notably, the findings from the deletion experiments involving CXCR4^+^ HSCs did not entirely correspond with those observed in CXCR4 ablation studies. Transplantation assays using the CXCR4⁺‐depleted HSC subset revealed markedly impaired reconstitution capacity, highlighting the critical role of CXCR4 in HSC homing and retention in the BM niche.^[^ ^64^, ^73^, ^74^ ^]^ While previous studies have shown that knocking out the *CXCR4* gene in HSCs or MPPs leads to severely decreased lymphopoiesis and a mild reduction in myeloid progenitors,^[^ ^66^, ^67^, ^75^, ^76^, ^77^ ^]^ our data indicate that the depletion of the CXCR4^+^ HSC subset promotes a shift in differentiation of remaining HSCs toward the myeloid lineage. Moreover, after restoring the CXCR4^+^ HSC subset, the MPN onset was largely postponed along with much more alleviated thrombocytosis and much restrained mutant cell expansion. Nevertheless, it is also possible that the reduction in CXCR4^+^ HSCs could be a consequence of the primary pathogenic events of driver mutations,^[^ ^65^, ^78^ ^]^ which impose a myeloid or Mk bias and thereby diminish the proportion of lymphoid‐biased CXCR4^+^ HSCs—a possibility that warrants further investigation.

In summary, it is possible that the diverse subsets of HSCs may have a compensatory relationship, whereby the hematopoietic lineage output may be regulated by a combination of distinct heterogeneous HSC subsets and the integrity of HSC heterogeneity may be of high necessity for hematopoietic homeostasis. This intriguing concept requires further investigation in future studies.

Despite the insights provided by our dataset, this study has limitations. The sample size and cell number, while informative, remain modest. Furthermore, the potential influence of co‐mutations, though partially mitigated by our analytical approach, warrants further investigation in future studies.

## 4 Experimental Section

### Materials Availability

This work did not yield any newly developed or unique reagents.

### Data and Code Availability

scRNA‐seq dataset have been submitted to the Genome Sequence Archive^[^ ^79^ ^]^ at the National Genomics Data Center,^[^ ^80^ ^]^ China National Center for Bioinformation/Beijing Institute of Genomics, Chinese Academy of Sciences, under accession numbers HRA005692 and HRA000669, accessible at https://ngdc.cncb.ac.cn/gsa‐human. The publicly available dataset GSE117826^[^ ^19^ ^]^ from Gene Expression Omnibus (https://www.ncbi.nlm.nih.gov/geo/) was also used in this paper. Any relevant information about the data reported in this study is available from the lead contact upon reasonable request.

### Experimental Model and Subject Details—Sample Collection

The study protocol was reviewed and approved by the Ethics Review Committee of the Blood Disease Hospital, Institute of Hematology, Chinese Academy of Medical Sciences (approval number: NKRDP2022003‐EC‐2). Every participant gave written informed consent before participation. For scRNA‐seq, bone marrow aspirates were harvested from thirteen *CALR*‐mutated, ten *MPL*‐mutated and five triple‐negative (TN) essential thrombocythemia (ET) patients. Diagnostic categorizations were in accordance with the criteria of World Health Organization (WHO). All enrolled patients were at the time of initial diagnosis and had not received prior therapy. Data pertaining to *JAK2* ^V617F^ patients and normal controls (NCs) were extracted from the prior study.^[^ ^16^ ^]^ Comprehensive clinical and laboratory characteristics of patients at the time of sampling are listed in Table S1 (Supporting Information). Additionally, the peripheral blood (PB) samples were obtained from NCs in the Institute of Hematology and Blood Diseases Hospital and umbilical cord blood (UCB) samples were from the biobank of the institute.

### Mouse Strains

Approval for all animal procedures was obtained from the Ethics Committee on Laboratory Animal Welfare at the Haihe Laboratory of Cell Ecosystem (approval number: HH‐DWLL‐NKRDP2022003‐2). *JAK2* ^V617F^ transgenic mice on a C57BL/6 background were utilized, and their generation and characterization have been described previously.^[^ ^81^ ^]^

*CALR* ^del52^ mice utilized in this study (C57BL/6 background) were characterized in detail and previously documented.^[^ ^50^ ^]^ *CALR* ^fl/fl^ or *CALR* ^fl/+^ mice were crossed with Mx1‐Cre^+^ mice to generate *CALR* ^floxed^ Mx1Cre^+^ mouse strain. Mice aged 6–8 weeks were injected with Poly(I:C) HMW (InvivoGen) at a dose of 10 mg kg^−1^ for 3 times every other day to carry the mutant recombined allele. Four weeks later, the peripheral blood was tested to confirm the MPN ET phenotype.

### Flow Cytometry

Human mononuclear cells (MNCs) were isolated from PB, BM, or UCB using Histopaque density gradient centrifugation (Sigma‐Aldrich). Then, the MNCs were stained with a BV510‐conjugated lineage panel containing CD3, CD14, CD16, CD19, CD20, and CD56, along with APC‐CD34, PE‐Cy7‐CD38, APC‐H7‐CD45RA, Percp‐Cy5.5‐CD123, and PE‐CXCR4 antibodies. For 3′‐TARGET‐seq, RT‐qPCR, and analysis of CXCR4⁺ HSC proportions in patients, HSCs characterized by the Lin^−^CD34⁺CD38^−^CD45RA^−^CD123^−^ phenotype were analyzed and isolated. For experimental validation, Lin^−^CD34⁺CD38^−^ or Lin^−^CD34⁺ HSPCs were sorted for Mk differentiation, CFU‐Mk assays, and related analyses.

Mice MNCs were harvested from tail blood, femurs, tibias, or spleen. Bone marrow MNCs were stained with APC‐eFluor780‐labeled lineage antibody cocktail targeting CD3e, CD4, CD8a, TER‐119, Gr‐1, CD11b, and B220, together with APC‐conjugated CD117 (c‐Kit), PE‐Cy7‐conjugated Sca‐1 antibodies. This staining strategy was used to identify and sort LSKs (Lin^−^Sca‐1^+^c‐Kit^+^) from WT and ET mice. CXCR4^+^ LSKs were additionally stained with PE‐conjugated CXCR4 antibody. For analyzing the reconstitution frequency after bone marrow transplantation, LT‐HSC was identified as Lin^−^Sca‐1^+^c‐Kit^+^CD34^−^CD135^−^, ST‐HSC as Lin^−^Sca‐1^+^c‐Kit^+^CD34^+^CD135^−^ and MPP as Lin^−^Sca‐1^+^c‐Kit^+^CD34^+^CD135^+^. The staining included APC‐eFluor780‐conjugated CD3e, CD4, CD8a, TER‐119, Gr‐1, CD11b, and B220, PE‐Cy7‐conjugated Sca‐1, BV605‐conjugated CD117 (c‐Kit), BV421‐conjugated CD135, and FITC‐ or APC‐conjugated CD34. MEP characterized by Lin^−^Sca‐1^−^c‐Kit^+^ CD34^−^CD16/32^−^, CMP by Lin^−^Sca‐1^−^c‐Kit^+^ CD34^+^CD16/32^lo^, and GMP by Lin^−^Sca‐1^−^c‐Kit^+^ CD34^+^CD16/32^hi^ were stained with APC‐eFluor780‐conjugated lineage antibodies (CD3e, CD4, CD8a, TER‐119, Gr‐1, CD11b, and B220), in combination with PE‐Cy7‐conjugated Sca‐1 or BV785‐conjugated Sca‐1, BV605‐conjugated CD117 (c‐Kit), FITC‐ or APC‐conjugated CD34, and Percp‐Cy5.5‐conjugated CD16/32 antibodies; MkP (Lin^−^Sca‐1^−^c‐Kit^+^CD150^+^CD41^+^) with APC‐eFluor780‐conjugated CD3e, CD4, CD8a, TER‐119, Gr‐1, CD11b, and B220, PE‐Cy7‐conjugated Sca‐1, BV605‐conjugated CD117 (c‐Kit), BV421‐conjugated CD150 and APC‐conjugated CD41; Mks (CD41^+^CD42d^+^) with APC‐conjugated CD41 and PE‐conjugated CD42d antibodies; B cells with Percp‐Cy5.5‐conjugated B220 antibody; T cells with PE‐Cy7‐conjugated CD3 antibody; Gr‐1^+^/CD11b^+^ cells with APC‐eFluor780‐conjugated Gr‐1 and CD11b antibodies; CD45.2^+^ cells with FITC‐, APC‐Cy7‐ or Percp‐Cy5.5‐conjugated CD45.2 antibody. Comprehensive antibody information can be found in Table S4c (Supporting Information).

After antibody incubation for 30 or 90 min in the dark and cell washing, flow cytometry was performed either by a FACS canto II flow cytometer (BD Biosciences), or a FACS Aria III flow cytometer (BD Biosciences).

### TARGET‐seq

The 3′‐TARGET‐seq, a method combining single‐cell transcriptomic profiling and mutation detection, was conducted with modifications based on a previously described protocol.^[^ ^16^, ^18^, ^82^ ^]^ First, the single HSCs from NCs and *CALR*‐mutated/*MPL*‐mutated/TN ET patients were lysed using a buffer containing RNase inhibitor (2 U, Invitrogen), Triton X‐100 (0.095 µL of 10%, Sigma‐Aldrich), barcode primer (1 pmol), dNTP Mix (5 nmol, Invitrogen) and nuclease‐free water (1.805 µL), followed by incubation at 72 °C for 3 min, as previously described.^[^ ^16^ ^]^ Second, reverse transcription of the lysis product was carried out in a mixture containing SuperScript II reverse transcriptase (50 U, Invitrogen), Superscript II first‐strand buffer (1 µL, Invitrogen), DTT (25 nmol, Invitrogen), RNase Inhibitor (5 U, Invitrogen), betaine (5 mmol, Sigma‐Aldrich), MgCl_2_ (0.03 mmol, Sigma‐Aldrich), and TSO (5 pmol). Third, a 22‐cycle of pre‐amplification was performed with 0.07–0.14 µL *CALR*‐ or *MPL*‐mutated specific genomic primers (Table S4, Supporting Information), 6.25 µL HiFi HotStart ReadyMix (Kapa Biosystems), 6.25 pmol 3′ P2, 1.25 pmol IS PCR primers in a total mixture media of 12.5 µL. Fourth, each amplicon from pre‐amplification was divided into two parts. One was used for the mutation detection as follows.

To detect *MPLW515L, MPLW515K, MPLW515A*, and *CALR*‐type 2 mutant cells, PCR (35 cycles) was performed with a reaction mixture containing 5 µL product from pre‐amplification, 0.5 µL of 1 U µL^−1^ KOD‐Plus‐Neo (TOYOBO), 2.5 µL of 10× KOD buffer, 2 µL of 2 mm dNTP, 2 µL of 25 mm MgSO4, 10.5 µL of nuclease‐free water, 1.5 µL of 2 µm target‐specific primers (*MPL*‐gDNA‐F/R or *CALR*‐gDNA‐F1/R1).

Then, 1 µL amplicon product derived from KOD‐Plus‐Neo genome amplification mix was extracted to perform TaqMan assays for ultimate genotype determination. For genotyping validity, standardized samples (standardized WT sample: amplicons from sequencing‐validated wild type allele; standardized MU sample: amplicons from sequencing‐validated mutant allele; standardized HETERO sample: amplicons mixed in a 1:3 ratio, equally, or in a 3:1 ratio from sequencing‐validated mutant allele and wild type allele) were made to titrate the moderate concentrations of TaqMan probes to achieve accurate and validated genotyping since the affinity between alleles and probes may differ. Over *CALR*‐type 2 genotyping, the usage of 0.3 µL 10 µm *CALR*‐type 2 MU probe and 0.3 µL 20 µm was implemented, meanwhile *MPLW515L*, *MPLW515K*, *MPLW515A* genotyping was performed with 0.3 ul 10 µm *MPLW515L* MU probe versus 0.3 µL 10 µm *MPL* WT probe, 0.3ul 20 µm *MPLW515K* MU probe versus 0.3 µL 20 µm *MPL* WT probe and 0.3ul 10 µm *MPLW515A* MU probe versus 0.3 µL 10 µm *MPL* WT probe, separately. 1 µL amplicon product, 0.6 µL indicated Taqman probes, 0.3 µL 10 µm F+R primer, 5 µL TransTaq‐T PCR SuperMix (TransGen Biotech) and 3.1 µL nuclease‐free water were mixed for TaqMan assays by the QuantStudio 5 real‐time PCR system (Thermo Fisher Scientific) through which the amplification curve of WT and MU alleles could be detected. If the detected differences between MU and WT curves were 3 cycles within, the amplicon derived cell would be determined as heterozygous. If not, the cell would be recognized as either WT or MU homozygous depending on the earlier amplified allele curve.

For detecting the *CALR*‐type 1 mutant cells, a *CALR*‐specific forward primer labeled with 6‐carboxyfluorescein (6‐FAM) were used for the amplification of mutation specific genomic DNA, followed by a fragment analysis in BGI.

The other part was subjected to the library construction by using STRT‐seq procedure, as described previously.^[^ ^16^ ^]^ First, amplified cDNA was pooled and sequentially purified with DNA Clean and Concentrator kit (Zymo Research) and AMPure XP magnetic beads (Beckman Coulter) successively. Then the purified 3′‐amplified cDNA was capped with index sequences with biotin modification by PCR, fragmented to a ≈300 bp length and captured with Dynabeads MyOne Streptavidin C1 beads (Invitrogen). Finally, the sequencing libraries were constructed using KAPA Hyper Prep Kits (Kapa Biosystems) and sequenced with an Illumina HiSeq 4000/NovaSeq 6000 sequencing instrument. Specific information on the above reagents was provided in Table S4c (Supporting Information).

### scRNA‐seq Data Preprocessing and Quality Control

Based on an 8‐bp cell‐unique barcode, sequencing reads were demultiplexed, and individual FASTQ files were generated for each cell. Subsequently, reads were mapped to the human GRCh38/hg38 reference genome with the HISAT2 aligner^[^ ^83^ ^]^ and annotated by Gencode V27 transcripts dataset. Alignment files were converted to .bam format using Samtools, and gene counts were obtained with StringTie.^[^ ^83^ ^]^ Quality control (QC) filtering accorded with the following criteria: library size > 50000 reads, percentage of reads mapped to the mitochondrial genome < 25%, and a minimum of 500 detected genes per cell (count > 0). Normalization and scaling of counts were performed using the Seurat R package,^[^ ^84^ ^]^ employing the LogNormalize method.

### Dimensionality Reduction and Cell Clustering

Seurat R package was used for downstream dimensionality reduction and cell clustering. A selection of two thousand highly variable genes (HVG) identified by “vst” method was employed for dimension reduction by principal‐component analysis (PCA) and the following cell clustering. Subsequently, the data was scaled and conducted by PCA. The first 10 components were utilized for uniform manifold approximation and projection (UMAP) and the visualization. Subsequent cell clustering was executed using the FindClusters function in Seurat with a resolution value of 0.4, yielding distinct cellular populations.

### Detection of Differentially Expressed Genes (DEGs) and Functional Gene Ontology (GO) Enrichment Analysis

Raw counts were first normalized by Seurat R package using LogNormalize method. To identify differentially expressed genes, a Wilcoxon rank‐sum test was implemented with Bonferroni correction as the default method for multiple testing adjustment. Significant genes were defined as having a logFold Change >0.25, minimum percentage of expressed cells >0.1 and *P* < 0.05. Then Gene Ontology was performed by DAVID tools^[^ ^85^, ^86^ ^]^ (https://david.ncifcrf.gov/)

### Gene Set Enrichment Analysis (GSEA)

GSEA was conducted using gene sets obtained from MSigDB (http://software.broadinstitute.org/gsea) and pertinent literature.^[^ ^45^, ^59^, ^60^ ^]^ Gene expression matrix, normalized through the Seurat R package, were utilized as the input data. A permutation number of 1000 and the “phenotype” permutation type were employed. The significance of enrichment scores was adjusted for multiple testing using the false discovery rate (FDR) method, with significance thresholds set at *P* < 0.05 and FDR < 0.25, in accordance with standard GSEA guidelines.^[^ ^87^ ^]^

### Construction of Lentiviral shRNA Vector and Cell Infection

To knock down gene expression, lentiviral vectors carrying gene shRNA were constructed. The shRNA targeting sequences for *STIM1* are as the following: 1) 5′‐CCTGGATGATGTAGATCATAA ‐3′ and 2) 5′‐ GAGGTGCAATATTACAACATCAAGA ‐3′; for *SREBF1*: 1) 5′‐ GCTGAATAAATCTGCTGTCTT ‐3′ and 2) 5′‐ CCAGAAACTCAAGCAGGAGAA ‐3′; for *SREBF2*: 1) 5′‐ CCTCAGATCATCAAGACAGAT ‐3′ and 2) 5′‐ GACCTGAAGATCGAGGACTTT ‐3′; for *FASN*: 1) 5′‐ GCTACGACTACGGCCCTCATT ‐3′ and 2) 5′‐ CATGGAGCGTATCTGTGAGAA ‐3′. The shRNA oligonucleotides were subcloned into either the pSIH1‐H1‐copGFP lentiviral vector (System Biosciences) or the pLKO.1‐puro shRNA expression vector (Addgene). The scrambled control shRNA was constructed following the manual recommendations. To produce lentivirus, 293T cells were transiently co‐transfected with 5 µg psPAX2 packaging plasmid, 3 µg pMD2.G envelope plasmid and 7 µg targeting shRNA plasmid by Lipofectamine 2000 (Invitrogen). Then packaging virus was harvested from the supernatant of transfected 293T cells.

To knock down gene expression, cells were infected with targeting shRNA or scrambled shRNA virus at a density of 6.5 × 10^5^ mL^−1^ and the medium was changed to fresh medium 12 h after infection.

### Cell Culture and Megakaryocyte Differentiation

MEG‐01 cells were cultured in RPIM 1640 (Gibco), supplemented with 10% FBS and 1% Penicillin Streptomycin (P/S), in a humidified incubator at 37 °C with 5% CO_2_. After virus infection, cells were expanded for protein extraction, which was used to perform western blot (WB) to verify the knockdown efficiency.

After virus infection, part of UCB HSPCs (Lin^−^CD34^+^) was harvested for RNA extraction, and the remaining cells were subjected to megakaryocyte differentiation. On days 0–6 of differentiation, the cells were cultured using StemSpan medium (Stem Cell Technologies) supplemented with recombinant cytokines, including recombinant human thrombopoietin (TPO) (50 ng mL^−1^, PeproTech), recombinant human stem cell factor (SCF) (20 ng mL^−1^, Prospec Bio), and recombinant human interleukin‐3 (IL‐3) (20 ng mL^−1^, Sigma‐Aldrich). Then on days 6–12, the culture medium was supplemented with 50 ng mL^−1^ TPO and 20 ng mL^−1^ IL‐11. The culture medium was refreshed every three days, meanwhile the proportion of CD41a and CD42b was detected using flow cytometry. Cell density was maintained ≈1 × 10^5^ cells mL^−1^.

### Colony Forming Unit Assays

FACS‐sorted total, CXCR4^+^, or CXCR4^−^ HSPCs (Lin^−^CD34^+^CD38^−^) or total, CXCR4^+^, or CXCR4^−^ HSPCs (Lin^−^CD34^+^) from PB or UCB, respectively were diluted in Iscove's Modified Dulbecco's Medium (IMDM, Gibco) containing 2% FBS to achieve a final cell concentration of 5000–10000 cells mL^−1^. The cells were subsequently suspended in MethoCult medium (STEMCELL Technologies) and cultured at 37 °C under 5% CO_2_ with at least 95% humidity. After expansion of 14 days, different colonies were identified and counted following the manufacturer's instructions.

For CFU‐Mk assays, FACS‐sorted UCB Lin^−^CD34^+^CD38^−^ HSPCs with *FASN*, *SREBF1*, *SREBF2*, or *STIM1* depletion were resuspended in IMDM (Gibco) at 1.1 × 10⁵ cells mL^−1^, mixed thoroughly with MegaCult‐C media and Collagen Solution (STEMCELL Technologies), and seeded according to the manufacturer's instructions. After 14 days of culture, CFU‐Mk colonies were fixed, stained, and examined. Additionally, CXCR4^+^Lin^−^CD34^+^CD38^−^ HSPCs from UCB and CXCR4^+^Lin^−^CD34^+^ HSPCs from PB were sorted by flow cytometry and subjected to CFU‐Mk assays.

### Intracellular Calcium Measurement

Lin^−^CD34^+^CD38^−^ HSPCs with *FASN*, *SREBF1*, or *SREBF2* depletion were loaded with Fluo‐4 AM (Beyotime; 4 µm, 0.02% Pluronic F‐127) in HBSS buffer containing 1.3 mm Ca^2^⁺, 1% FBS, and 2.5 mm probenecid, incubated at 37 °C for 30 min, washed three times with 3–5 min resting, and further incubated dye‐free for 10–15 min to complete de‐esterification. Intracellular Ca^2^⁺ level was then quantified on a FACS canto II flow cytometer (BD Biosciences).

### Quantitative Real‐Time PCR

Total RNA was isolated using TRIzol reagent, and its concentration and purity were assessed with a NanoDrop 2000 spectrophotometer. Subsequently, cDNA was generated with the Hifair III Reverse Transcription Kit (Yeason) following the manufacturer's protocol. RT‐qPCR was performed with the SYBR Green PCR Reagent Kit (Applied Biosystems) using the 0.1‐QuantStudio 5 instrument (Thermo Fisher Scientific).

### Western Blot

MEG‐01 cells were briefly washed with PBS and centrifuged at 300 g for 5 min. The cell pellets were lysed on ice for 15 min using RIPA lysis buffer (Solarbio), fortified with protease and phosphatase inhibitors. The lysates were spun at 12,000 g for 10 min at 4 °C, after which the supernatant fractions were collected. Protein concentrations were quantified using the bicinchoninic acid (BCA) reagent (Thermo Fisher). Protein samples were subjected to SDS–PAGE, followed by immunoblotting with anti‐SREBF1, anti‐SREBF2, anti‐FASN, and anti‐STIM1 (Proteintech), anti‐α‐tubulin (Abcam), and HRP‐conjugated goat anti‐rabbit or anti‐mouse IgG (H+L) (Proteintech). Blots were detected using chemiluminescence reagents (Yeasen), and images were processed using ImageJ software.

### Cell Cycle Analysis

cKit^+^ progenitor cells were enriched via CD117 microbeads (Miltenyi Biotec), following the recommended instructions, and the enriched cKit^+^ cells were fixed and permeabilized using the BD IntraSure kit. Then cells were stained by FITC‐conjugated Ki67(Invitrogen) and Hoechst 33342(Solarbio), and analyzed by the flow cytometer (BD Biosciences) mentioned before.

### In Vitro Liquid Culture Assays

For LSKs liquid culture assays, FACS‐sorted 400 LSKs were grown in 200 µL cytokine‐enriched medium as described previously by Passegue ´ lab,^[^ ^51^ ^]^ which is Iscove's modified Dulbecco's media (IMDM) as the basic medium along with FBS (5%, Sigma‐Aldrich), P/S (1%, Gibco), L‐glutamine (2 mm, Gibco), non‐essential amino acids(0.1 mm, Gibco), sodium pyruvate(1 mm, Gibco), and 2‐mercaptoethanol(50 mm, Gibco). The medium was further supplemented with cytokines, including 25 ng mL^−1^ SCF (PeproTech), 25 ng mL^−1^ TPO (PeproTech), 25 ng mL^−1^ Flt3‐L (PeproTech), 25 ng mL^−1^ IL‐11 (PeproTech), 10 ng mL^−1^ IL‐3 (PeproTech), 10 ng mL^−1^ GM‐CSF (ABclonal) and 4 U mL^−1^ EPO (ESPO 3000). After 72 h incubation, the colony images were captured via high‐content analysis system (Perkinelmer Operetta CLS) and cells were counted, then 400 cells were extracted and re‐seeded in 200 µL cytokine‐enriched medium as the abovementioned. After a second 72 h incubation, colony images were captured and cell counts were recorded again.

### Serial Replating Assays

First, FACS‐sorted LSKs, CXCR4^+^ LSKs, and CXCR4^−^ LSKs were subjected to MethoCult media (Stemcell) for colony‐forming assays according to the manufacturer's instruction. After 12 days of culture, the colony numbers are quantified, and then the cells were collected and washed with IMDM to eliminate the MethoCult media for three times. 20000 cells were thereafter replated in MethoCult media for another 12 days incubation. All these procedures proceeded for 5 times, and the quantified colony‐forming units (CFUs) were displayed as the mean of at least three independent determinations.

### Intra‐BM Transplantation

To investigate whether direct deletion of the C5 subpopulation promotes MPN development, 3000 LSKs, CXCR4^+^ LSKs, or CXCR4^−^ LSKs (CD45.2) were delivered via intra–bone marrow injection into lethally irradiated CD45.1 mice, accompanied by 8 × 10⁵ bone marrow mononuclear cells (CD45.1) to facilitate hematopoietic reconstitution. To verify whether supplementation of the C5 subpopulation affects disease progression, 2000 LSKs, CXCR4^+^ LSKs, or CXCR4^−^ LSKs from GFP transgenic mice (CD45.2) co‐expressing GFP and 2000 LSKs from *JAK2* ^V617F^ ‐ET mice (CD45.2) were combined with 7x10^5^ BMNCs (CD45.1) and injected directly into the bone marrow of lethally irradiated CD45.1 recipient mice.

At 4‐week intervals post‐transplantation, tail‐vein blood samples were obtained to monitor hematopoietic reconstitution by assessing donor chimerism in peripheral blood cells. After 20–24 weeks, all mice were euthanized, and BM and spleen MNCs were isolated by flushing the femurs, tibias and by mechanically dissociating spleen of each mouse. Then hematopoietic cells were identified using various antibody combinations as previously documented.^[^ ^88^ ^]^ H&E staining of mouse femurs was done by the pathology department.

### Statistical Analysis

Statistical analyses were conducted using R software (v4.1.3; R Foundation for Statistical Computing, 2022) or GraphPad Prism (v9.5.1; GraphPad Software). Pathway activity scores and other continuous variables were compared using the Wilcoxon rank‐sum test or one‐way ANOVA followed by Dunn's, Dunnett's or Tukey's post hoc test, as appropriate. Correlation analyses were conducted using Pearson's method with Benjamini–Hochberg (BH) correction for multiple comparisons. Statistical significance was denoted as follows: *P < 0.05, **P < 0.01, ***P < 0.001. The specific statistical test applied in each experiment was indicated in the corresponding figure legend.

### Ethics Approval Statement

This work was approved by Ethics Committee on Laboratory Animal Welfare of the Haihe Laboratory of Cell Ecosystem, and Ethics Review Committee of the Blood Disease Hospital, Chinese Academy of Medical Sciences (Institute of Hematology, Chinese Academy of Medical Sciences).

### Patient Consent Statement

Informed consent was obtained from all patients.

## Conflict of Interest

The authors declare no conflict of interest.

## Author Contributions

J.T., D.W., H.S., T.S., Y.Z., and W.G. contributed equally to this work. L.S., L.Z., and J.T. designed and supervised the experiments. J.T., D.W., H.S., T.S., Y.Z., and W.G. performed the experiments and bioinformatics analysis. L.L., Y.Z., Y.L., X.J., R.F., and M.J. optimized the mouse model and collected the blood and bone marrow samples. J.G., J.M., C.G., and A.Z. also collected the blood and bone marrow samples. Z.X., E.J., R.Y., and S.M. interpreted the results. J.T., D.W., H.S., and L.S. wrote the manuscript.

## Supporting information



Supporting Information

Supporting Information

Supplemental Table 1

Supplemental Table 2

Supplemental Table 3

Supplemental Table 4

## Data Availability

Single‐cell RNA‐seq data are available at Genome Sequence Archive (GSA) under the accession numbers HRA005692 and HRA000669.
