# Supplementary material for: Sabotaged Integral HSC Heterogeneity Underlies Essential Thrombocythemia Development
Source: Adv Sci (Weinh). 2025 Nov 21;13(7):e05249. doi: 10.1002/advs.202505249 (PMC12866761; doi:10.1002/advs.202505249)
Supplement: Supplementary file 1 — Supporting Information [file ADVS-13-e05249-s001.docx]

**Figure S1. *MPL*-mutated HSCs in ET are characterized by prominent metabolic disturbances.**

(A) Pie chart depicting the accuracy of genotype validation for *MPL*. TRUE, the mutated/non-mutated genotype was correctly identified; FALSE, the mutated/non-mutated genotype was incorrectly identified; UnID, the genotype of the cell could not be determined.

(B) GSEA plots showing the enrichment of Hallmark IFNα response and IFNγ response in *MPL*-mutated (*MPL*-mu) vs *MPL*-non-mutated (*MPL*-non) HSCs from ET patients. IFNα, interferon-alpha; IFNγ, interferon-gamma.

(C) Violin plots illustrating the log2 normalized expression of *STAT1, STAT3,* and *STAT5A* in *MPL*-mutated, and *MPL*-non-mutated HSCs.

(D) GSEA plot showing Hallmark cholesterol homeostasis pathway enrichment in *MPL*-mutated vs *MPL*-non-mutated.

(E) The bar graph showing the top 10 predicted TFs which may regulate the core genes implicated in cholesterol homeostasis (*MPL*-mutated vs *MPL*-non-mutated). TF, transcription factor.

(F) Dot plot exhibiting the expression of genes associated with cholesterol homeostasis in *MPL*-mutated and *MPL*-non-mutated ET HSCs. The dot color and size indicate Aver. Exp and Per. Exp, respectively.

(G) Western blot (WB) analysis and subsequent quantification confirmed the knockdown efficiency of fatty acid metabolism-related genes, including *SREBF1, SREBF2,* *FASN*, and *STIM1* in MEG-01 cells (n=3).

(H) Schematic illustration (top) of Mk differentiation from UCB HSPCs (Lin^-^CD34^+^) with gene knockdown; bar graphs (bottom) indicating the validation of gene knockdown efficiency in UCB HSPCs (Lin^-^CD34^+^) using RT-qPCR, including *SREBF1, SREBF2,* and *FASN* (n=3).

(I) Representative flow cytometry plots showing the percentage of Mk (CD41a^+^CD42b^+^) differentiated from UCB HSPCs (Lin^-^CD34^+^).

(J) Bar graphs illustrating a significant reduction in the proportion of Mk differentiation (CD41a⁺CD42b⁺) at days 9 and 12 following the knockdown of *SREBF1, SREBF2, and FASN* in UCB HSPCs (Lin^-^CD34^+^) (n=3-4).

(K) Contour plots showing the correlation between the Mk priming module and the SOCE module in *MPL*-mutated (left) and *MPL*-non-mutated (right) HSCs. The horizontal and vertical lines indicate the 75th percentile. Both the colored bands and marginal rugs show the distribution of each axis.

(L-M) Relative expression of *GRP78* and *ATF4* (L), and *STIM1* (M) in UCB HSPCs (Lin^-^CD34^+^CD38^-^) after knockdown of *FASN, SREBF1,* and *SREBF2*. Expression levels were normalized to the scramble control (n=3-4).

(N) Bar graph indicating the validation of *STIM1* knockdown efficiency in UCB HSPCs (Lin^-^CD34^+^) using RT-qPCR (n=3).

(O) Bar charts illustrating a marked decrease in the proportion of Mk differentiation (CD41a⁺CD42b⁺) at days 9 and 12 after *STIM1* knockdown in UCB HSPCs (Lin^-^CD34^+^) (n=3-4).

(P) Bar graphs showing the relative numbers of BFU-E and CFU-GM colonies formed from UCB-derived HSPCs (Lin⁻CD34⁺) following knockdown of *SREBF1, SREBF2, FASN*, and *STIM1* (n=6).

In B and D, significantly enriched pathways were determined by normal *P* <0.05 and FDR<0.25. In G-H, J, and L-P, data are presented as mean ± SEM. One-way ANOVA followed by Dunnett’s multiple comparisons test was used to calculate *P*-values. **P* < 0.05, ***P* < 0.01, ****P* < 0.001; ns, not significant.

**Figure S2.** ***CALR*-mutated HSCs exhibit clear proliferative signatures.**

(A) Pie chart depicting the accuracy of genotype validation for *CALR* mutation. TRUE, the mutated/non-mutated genotype was correctly identified; FALSE, the mutated/non-mutated genotype was incorrectly identified; UnID, the genotype of the cell could not be determined.

(B) GSEA plot showing the enrichment of Hallmark unfolded protein response genes in *CALR*-mutated (*CALR*-mu) vs *CALR*-non-mutated (*CALR*-non) HSCs.

(C) Violin plots showing the expression of four key genes associated with unfolded protein response (UPR) in *CALR*-non-mutated and *CALR*-mutated HSCs.

(D) GSEA plot (left) showing enrichment of regulation of cholesterol metabolism (GO:0008203) and dot plot (right) indicating the expression of related genes in *CALR*-mutated and *CALR*-non-mutated HSCs.

(E) Nightingale rose chart showing the top 50 enriched gene ontology (GO) terms from the analysis of differentially expressed genes (DEGs) identified from the comparison of *CALR*-mutated and *CALR*-non-mutated HSCs. Terms related to cell cycle and DNA replication are highlighted in red.

(F) Experimental design for liquid culture assays on *CALR* ^del52^ and *CALR* ^wt^ LSKs.

(G) Radar chart depicting the relative expression of mitochondrial respiratory chain complex genes in NC (green), *CALR*-non-mutated (blue), and *CALR*-mutated (orange) HSCs. The log2 expression of genes associated with the CI, CII, CIII, CIV, and CV protein complex in NC HSCs was normalized to 0; data from *CALR*-mutated and *CALR*-non-mutated HSCs were presented as fold change values.

(H-I) Box plots illustrating the expression of ATP synthesis (as listed in Table S3) (H), Hallmark oxidative phosphorylation, Hallmark glycolysis, and Hallmark fatty acid metabolism (I) in *CALR*-mutated and *CALR*-non-mutated HSCs.

(J) Bar charts illustrating the diminished proliferation (left) and differentiation (right) of MEG-01 cells upon treatment with the mTOR inhibitor rapamycin (n=5). Data are presented as the mean ± SEM. One-way ANOVA followed by Dunnett’s multiple comparisons test was used to calculate *P*-values. **P* < 0.05, ***P* < 0.01, ****P* < 0.001; ns, not significant.

(K) Uniform manifold approximation and projection (UMAP) plot displaying data from a single-cell dataset from a prior study^[9d]^, which distinguished *CALR*-mutated from *CALR*-non-mutated HSPCs by Genotyping of Transcriptomes (GoT) from bone-marrow-derived CD34^+^ cells of ET patients (GSE117826).

(L) Dot plot showing the expression of signature genes in each cluster defined in (K). The dot color and size indicate the average expression level (Aver. Exp) and percentage of cells expressing a given factor (Per. Exp), respectively.

(M) Feature plot depicting the expression of *AVP* and *HOPX*, two signature genes of human bone marrow (BM) HSPCs.

(N) Box plots showing the expression of Mk priming^[24]^, Reactome cell cycle (R-HSA-1640170), Hallmark OXPHOS, Hallmark fatty acid metabolism, and ATP synthesis (as listed in Table S3) in *CALR*-type 2 vs *CALR*-type 1 mutant HSPCs from the GSE117826 dataset^[9d]^. Both median (black bar) and mean (red diamond) expression levels are shown.

In B and D, significantly enriched pathways were determined by normal *P* <0.05 and FDR<0.25. In H, I, and N, *P*-values were determined by Wilcoxon rank-sum test. **P* < 0.05, ***P* < 0.01, ****P* < 0.001; ns, not significant.

**Figure S3. Biological processes shared by HSCs with distinct driver mutations.**

(A) Bar plot showing top GSEA enriched pathways in *JAK2* ^V617F^-mutated (*JAK2* ^V617F^ -mu) comparing to *JAK2* ^V617F^*-*non-mutated ET HSCs (*JAK2* ^V617F^-non).

(B) Bar graphs depicting the enriched hallmark gene sets in *CALR*-mu vs *MPL*-mu, *CALR*-mu vs *JAK2* ^V617F^-mu, *JAK2* ^V617F^-mu vs *MPL*-mu HSCs.

(C) Box plots displaying the score of genes associated with Hallmark TGFβ signaling (left) and Hallmark MYC signaling (right) among NC, *CALR*-non, *JAK2* ^V617F^-non and *MPL*-non HSCs, both median (black bar) and mean (red diamond) expression levels are shown. *P*-values were determined by Wilcoxon rank-sum test followed by Dunn’s multiple comparisons test. **P* < 0.05, ***P* < 0.01, ****P* < 0.001; ns, not significant.

(D) Bar graphs of enriched hallmark gene sets in *CALR*-non vs *MPL*-non, *CALR*-non vs *JAK2* ^V617F^-non, *JAK2* ^V617F^-non vs *MPL*-non.

In A, B, and D, significantly enriched pathways were determined by normal *P* <0.05 and FDR<0.25. The bar color and length respectively indicate –log10 (*P* value) and normalized enrichment score (NES).

**Figure S4. Identification of a stem cell subset exhibiting malignant features in TN ET HSCs.**

(A) GSEA plot (left) and box plot (right) showing the upregulation the of GOBP inflammatory response (GO:0006954) in C6 and vs HSC subsets. *P*-values were determined by Wilcoxon rank-sum test in the box plot. **P* < 0.05, ***P* < 0.01, ****P* < 0.001; ns, not significant.

(B) The expression of genes correlated with inflammation (GO:0006954) in each cluster.

(C) GSEA plot showing the enrichment of Hallmark inflammatory response gene set in the C6 of TN ET vs NC HSCs.

(D) GSEA plot depicting the enrichment of the Reactome IFN alpha-beta signaling (R-HSA-909733) in the C6 of TN ET vs NC HSCs.

In A, C, and D, significantly enriched pathway was determined by normal *P* <0.05 and FDR<0.25.

**Figure S5. The C5 cluster is largely absent from all ET subtypes.**

(A) UMAP depicting the clustering of all HSCs from NCs and *JAK2* ^V617F^-, *CALR*-, and *MPL-*mutated, and TN ET patients. HSCs were clustered into seven clusters (C0-C6).

(B) Dot plot showing the expression of signature genes in each cluster. The dot color and size indicate the average expression level (Aver. Exp) and percentage of cells expressing a given factor (Per. Exp), respectively.

(C) The frequency of HSCs in each cluster across NCs and distinct subtypes of ET patients.

(D) Uniform manifold approximation and projection (UMAP) plot (left) displaying NC HSCs mapped onto a 10X single-cell dataset of CD34^+^ HSPCs (n = 12229 cells total) from the bone marrow of two healthy donors (unpublished). Dot plot (middle) showing the expression of signature genes in each cluster defined in the left panel. The dot color and size indicate the average expression level (Aver. Exp) and percentage of cells expressing a given factor (Per. Exp), respectively. Subcluster C5 is highlighted in red in the UMAP (right).

(E) PHATE (Potential of Heat-diffusion for Affinity-based Trajectory Embedding) embedding of single-cell transcriptomes of cells in Figure S5D (n = 12229 cells total), colored by cell types (green: HSPC1-2, blue: lymphoid lineages, red: megakaryocyte and erythroid lineages, purple: myeloid lineages). C0-C6 subclusters of NC HSCs were mapped onto the branches of trajectory.

(F) Box plot comparing the expression levels of core enrichment genes from the GO regulation of lymphocyte proliferation pathway (GO:0050670) of C5 and other clusters in NC HSCs; both median (black bar) and mean (red diamond) expression levels are shown. Wilcoxon rank-sum test was used to calculate *P*-values. **P* < 0.05, ***P* < 0.01, ****P* < 0.001; ns, not significant.

**Figure S6. HSCs lacking CXCR4^+^ subset exhibit pronounced myeloid lineage bias**

(A) Outlines of the colony-forming unit (CFU) assay experimental scheme. Total, CXCR4^+^, or CXCR4^-^ HSPCs (Lin^-^CD34^+^) were FACS-sorted from the peripheral blood (PB) of NCs and then subjected to a CFU assay. The BFU-E, CFU-GM, and Mk colonies were identified and counted after 14 days of culture; the representative images of BFU-E, CFU-GM, and Mk colonies were shown on the right.

(B) The relative numbers of BFU-E, CFU-GM, total myeloid, and Mk colonies from total, CXCR4^+^, and CXCR4^-^ HSPCs (Lin^-^CD34^+^) isolated from PB. Each dot represents a biological replicate. The number of colonies generated from total HSCs was normalized to 1 (n=12-20). Data are presented as the mean ± SEM. One-way ANOVA followed by Tukey’s multiple comparisons test was used to calculate *P*-values. **P* < 0.05, ***P* < 0.01, ****P* < 0.001; ns, not significant.

**Figure S7. CXCR4^+^ HSCs transplantation delays MPN onset**

(A) Bar graphs displaying the relative numbers of BFU-E, CFU-GM, and total myeloid colonies formed in the first replating assay (n=5).

(B) Line graph showing the frequency of competitor-derived (CD45.2^+^) cells in tail blood of recipient mice at 4-week intervals until 24 weeks post-transplantation (n=5-11). The blue, red, and black lines indicate mice receiving CXCR4^+^, CXCR4^-^, or total LSKs respectively. Data are presented as the mean ± SEM.

(C) Peripheral blood cell count in recipient mice were measured at four-week intervals (n=5-11). Data are presented as the mean ± SEM. PLT, platelets; HGB, hemoglobin; WBC, white blood cells; HCT, hematocrit.

(D) Flow cytometry gating strategy used to identify the LSK (Lin^-^Sca-1^+^c-Kit^+^), LT-HSC (Lin^-^Sca-1^+^c-Kit^+^CD34^-^CD135^-^), ST-HSC (Lin^-^Sca-1^+^c-Kit^+^CD34^+^CD135^-^), MPP (Lin^-^Sca-1^+^c-Kit^+^CD34^+^CD135^+^), CMP(Lin^-^Sca-1^-^c-Kit^+^CD34^+^CD16/32^lo^), GMP (Lin^-^Sca-1^-^c-Kit^+^CD34^+^CD16/32^hi^), MEP (Lin^-^Sca-1^-^c-Kit^+^CD34^-^CD16/32^-^) and MkP (Lin^-^Sca-1^-^c-Kit^+^CD150^+^CD41^+^) subsets from the BM of mice post-transplantation.

(E) Bar plots displaying the frequency of competitor-derived LT-HSC, ST-HSC, MPP, CMP, GMP, and MEP in the BM of recipient mice transplanted with total, CXCR4^+^, and CXCR4^-^ LSKs (n=5-11).

(F) Peripheral blood cell count in recipient mice were measured at four-week intervals (n=6-9). Data are presented as the mean ± SEM. HGB, hemoglobin; WBC, white blood cells; HCT, hematocrit.

(G) Representative HE staining of recipient mouse bone marrow, with arrows indicating the presence of Mks.

(H) Bar charts depicting the proportions of MkPs and Mks in the bone marrow (BM), and Mks in the spleen following supplementation with CXCR4^+^ LSKs(n=6-9).

(I) Bar graphs showing that supplementation with CXCR4^+^ LSKs diminishes the stemness of *JAK2^V617F^*- LSKs and impairs their differentiation potential toward myeloid and megakaryocytic lineages (n=6-9).

In A, E, H, and I, data are presented as the mean ± SEM. One-way ANOVA followed by Tukey’s multiple comparisons test was used to calculate *P*-values. **P* < 0.05, ***P* < 0.01, ****P* < 0.001; ns, not significant.
