# Supplementary figures and images for: Sabotaged Integral HSC Heterogeneity Underlies Essential Thrombocythemia Development
Source: Adv Sci (Weinh). 2025 Nov 21;13(7):e05249. doi: 10.1002/advs.202505249 (PMC12866761; doi:10.1002/advs.202505249)

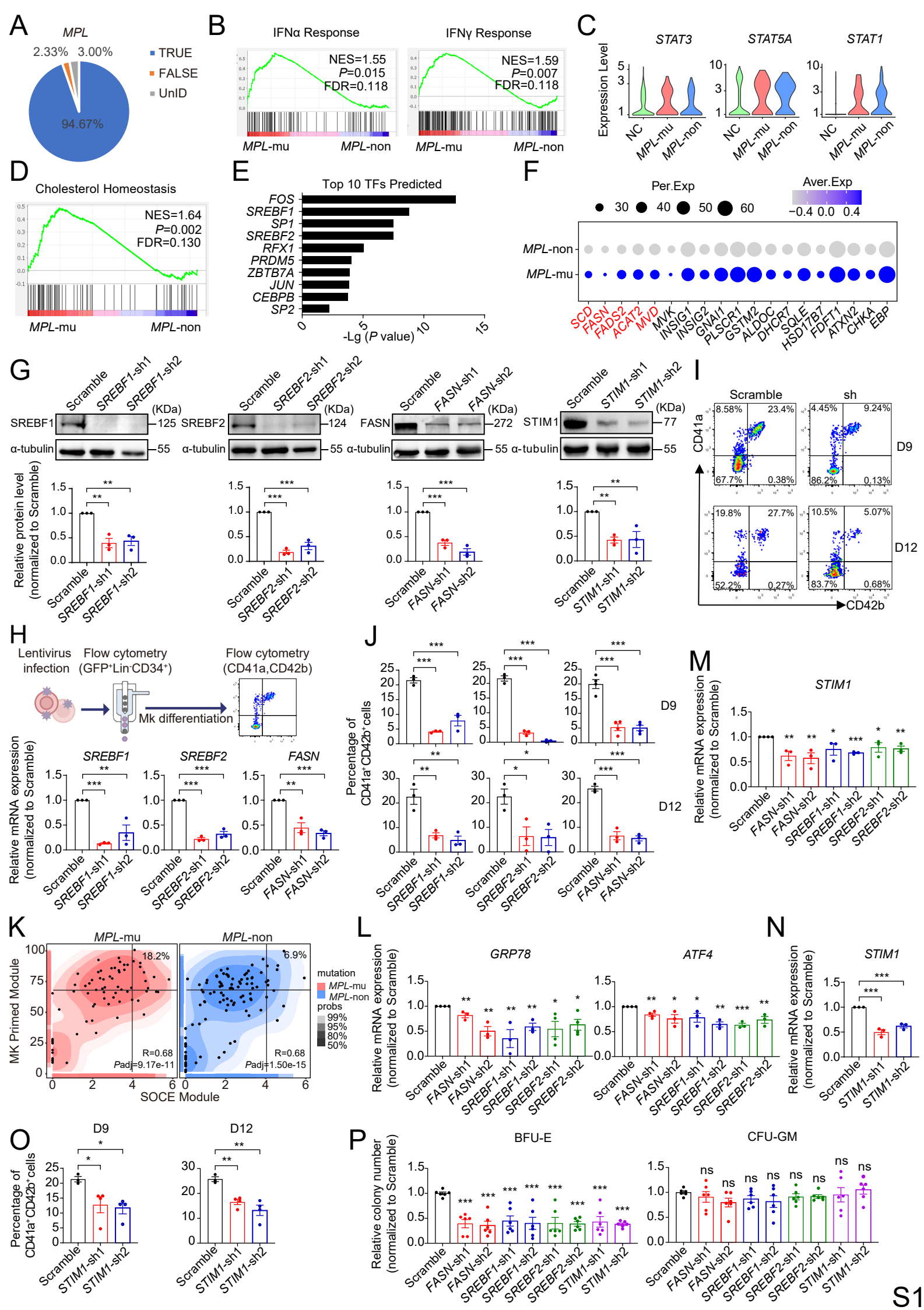

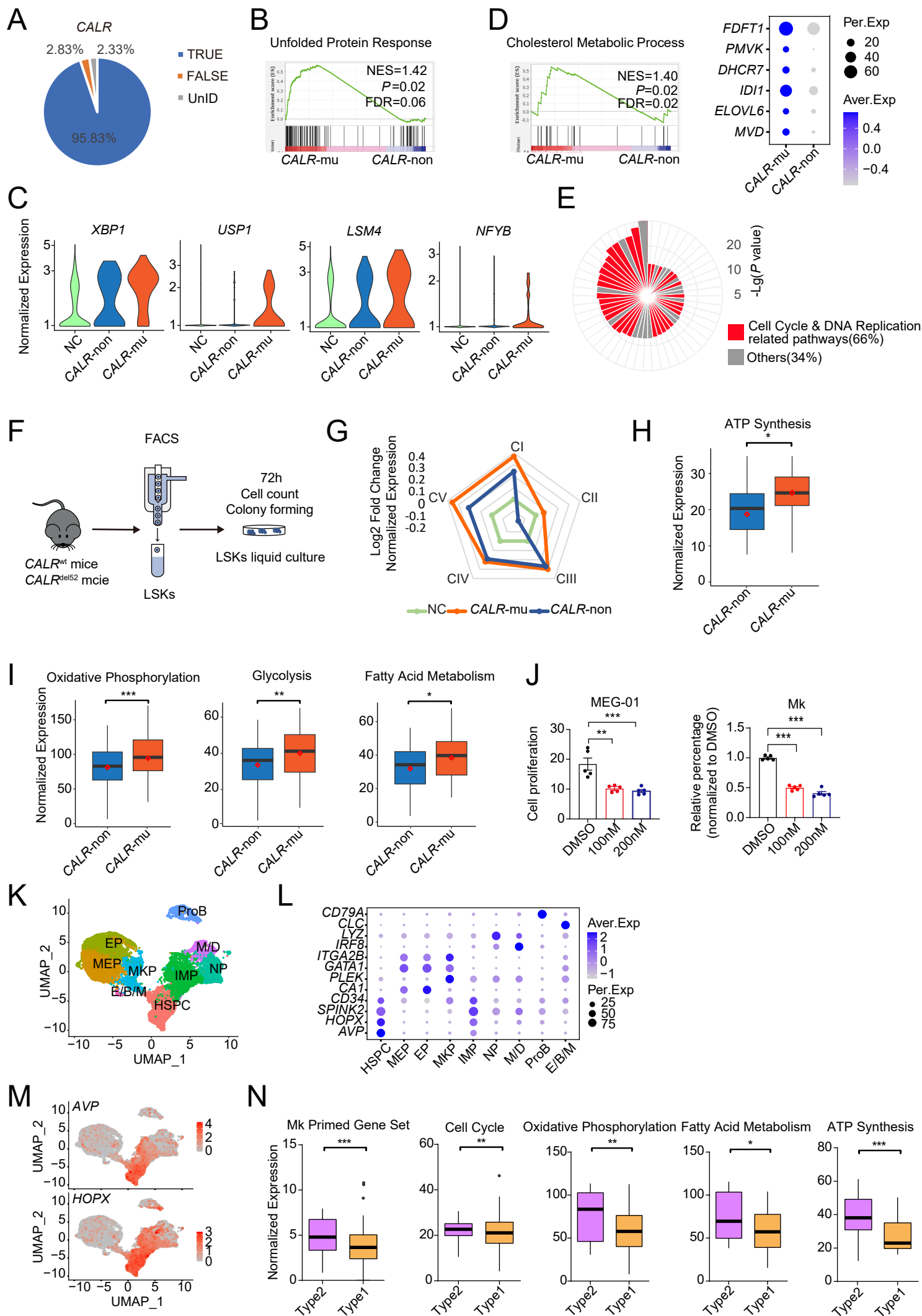

A

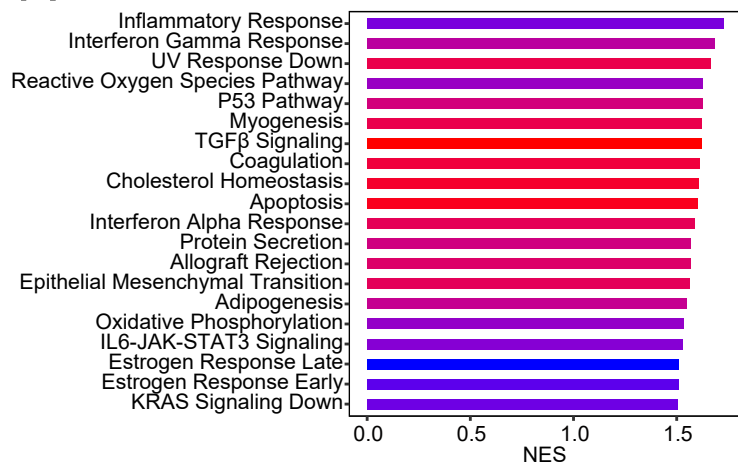

B

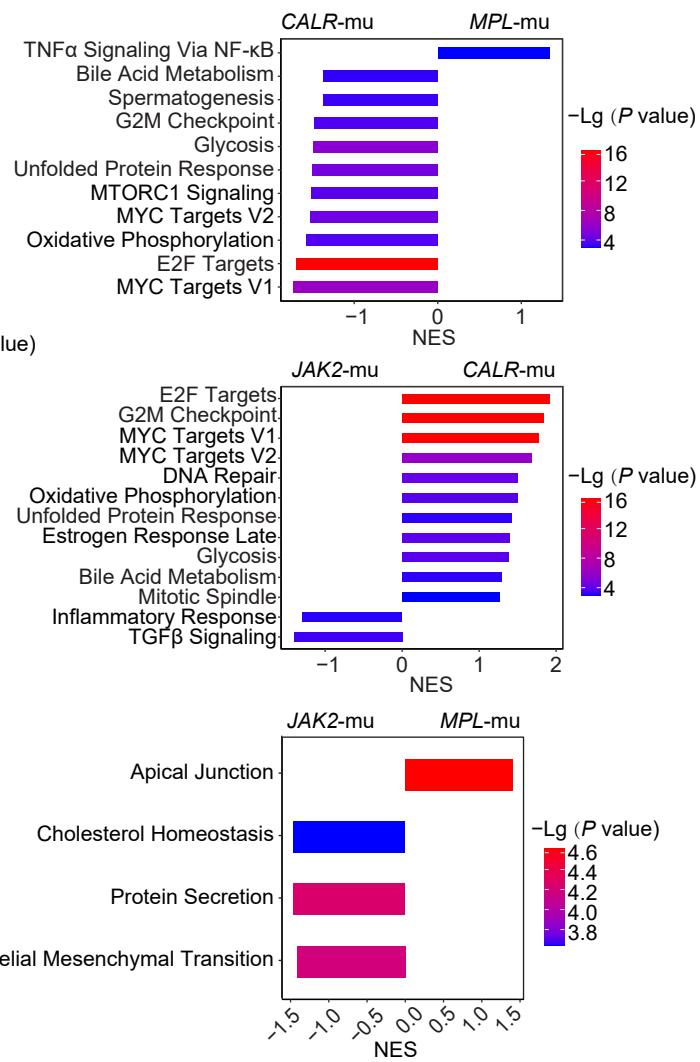

C

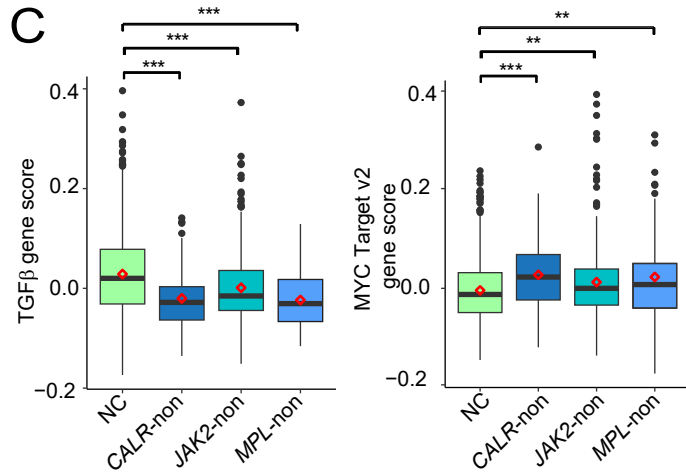

D

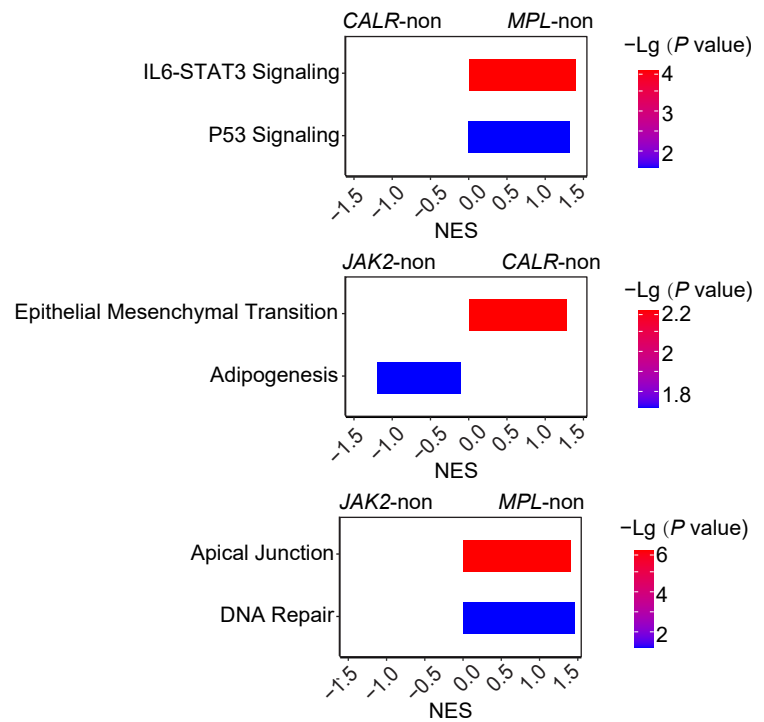

A

## Regulation of Inflammatory Response

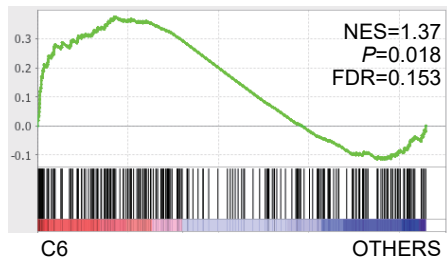

B

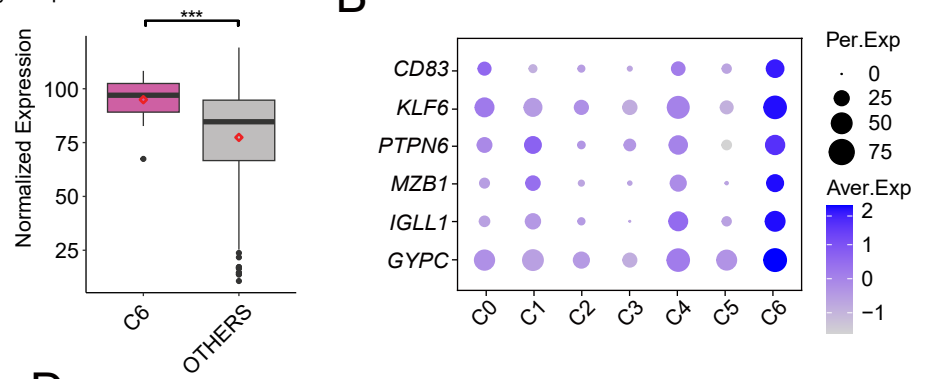

C

## Inflammatory Response

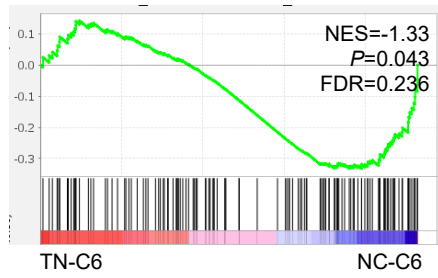

D

IFN $\alpha$  Signaling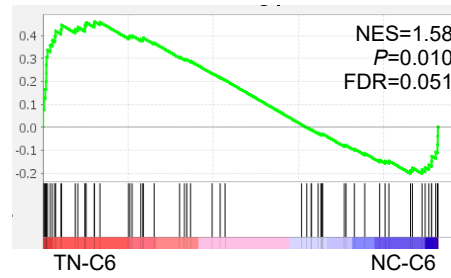

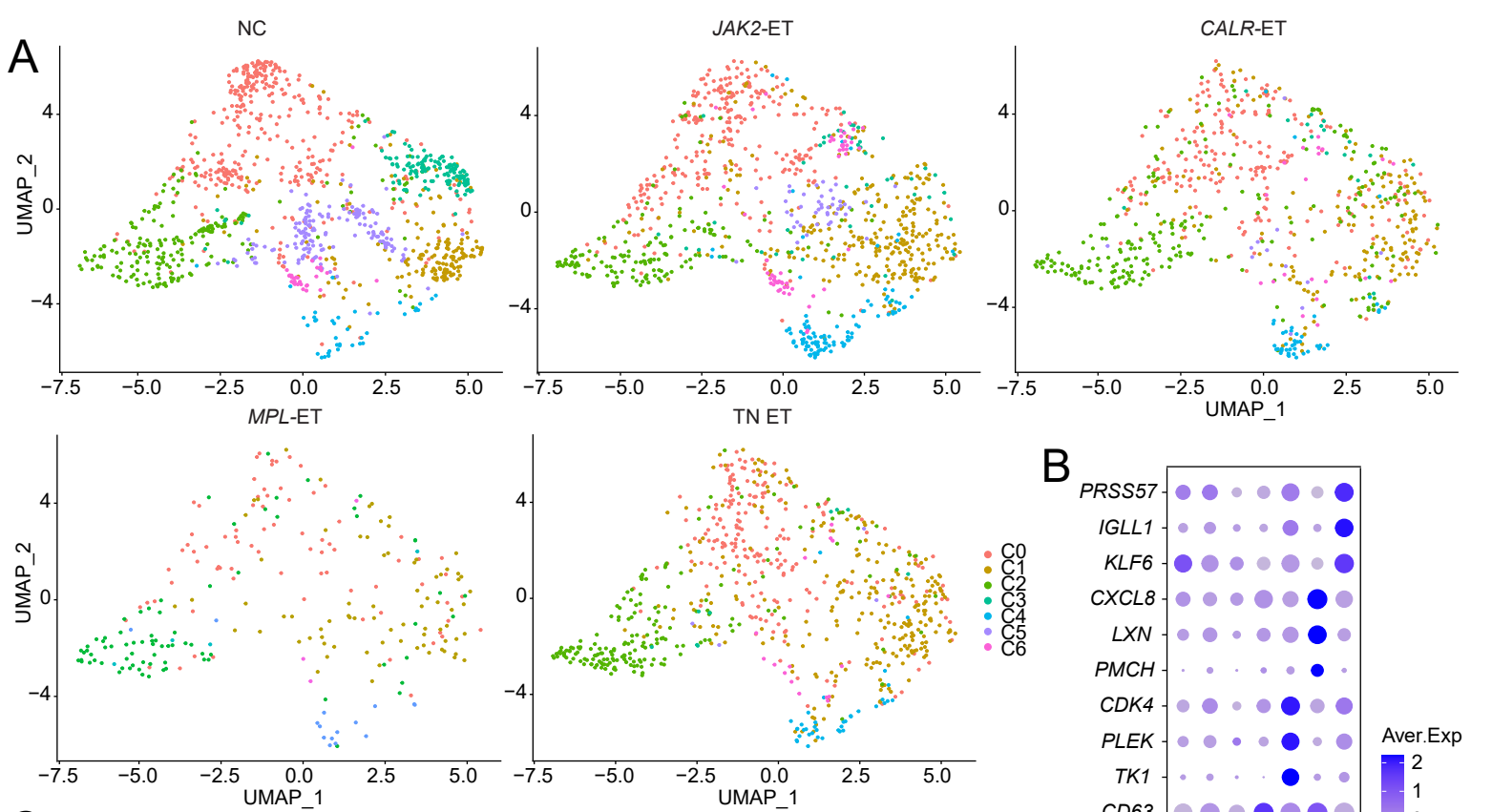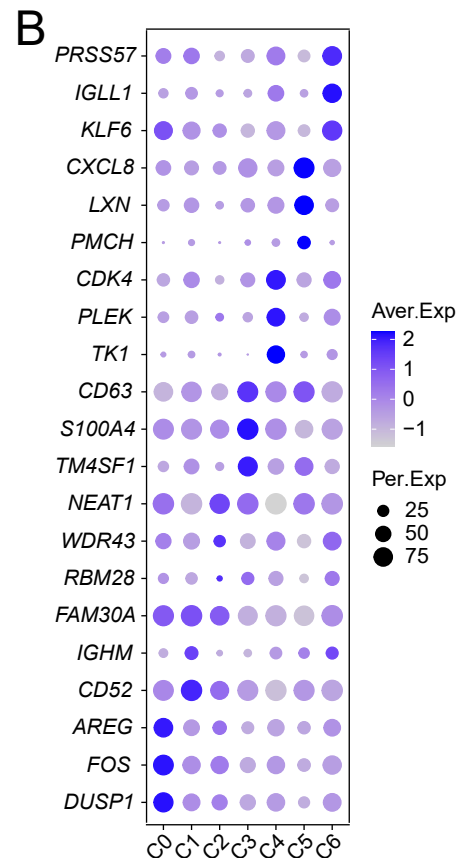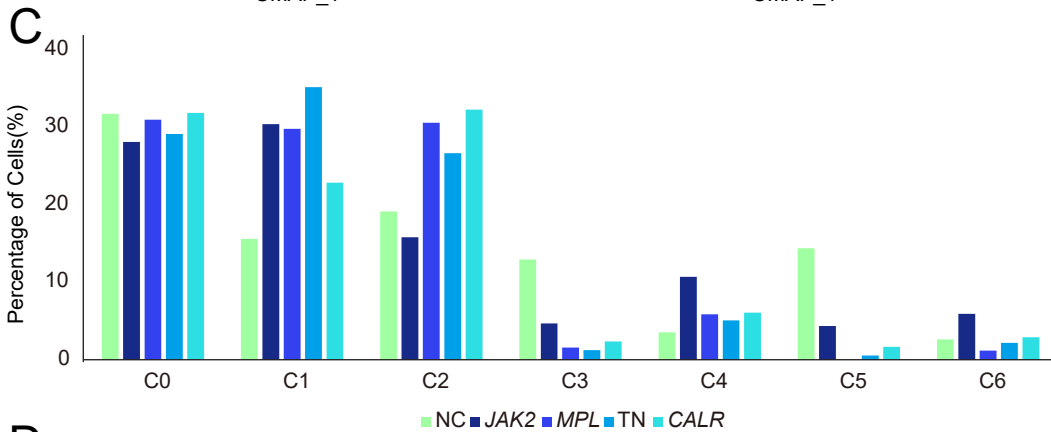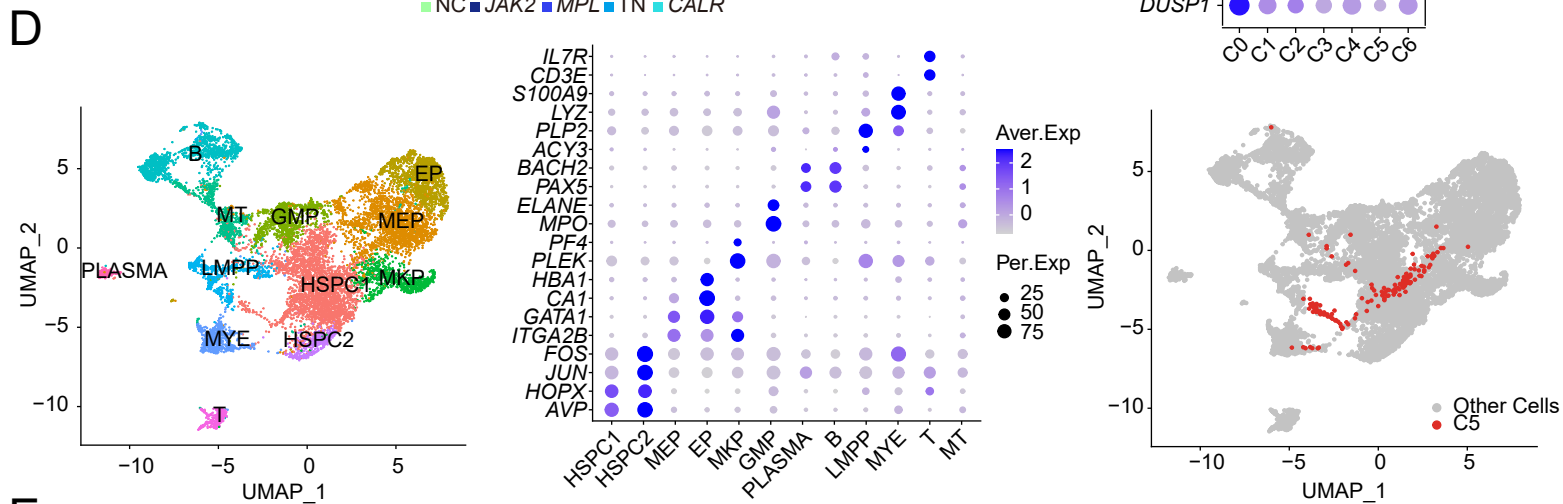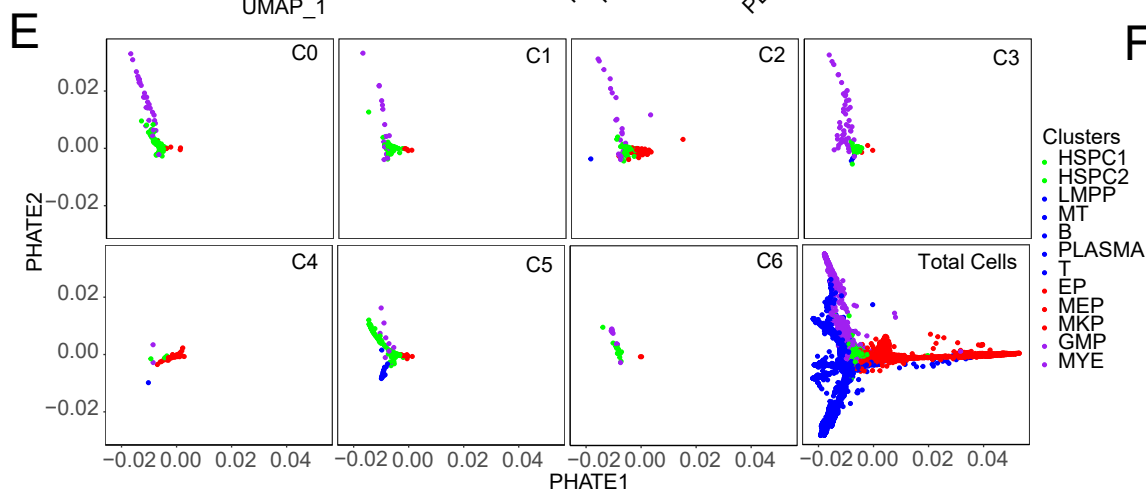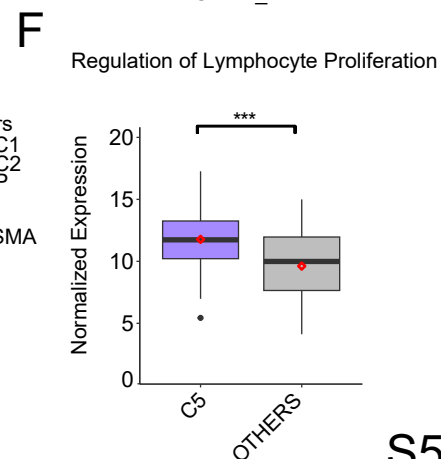

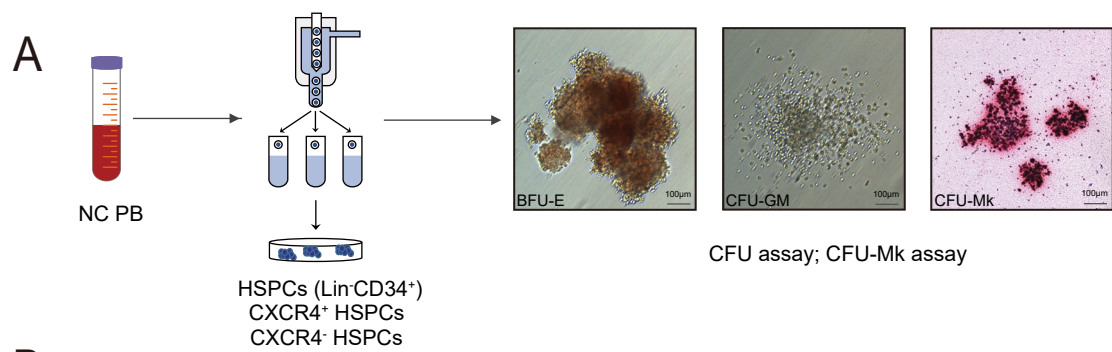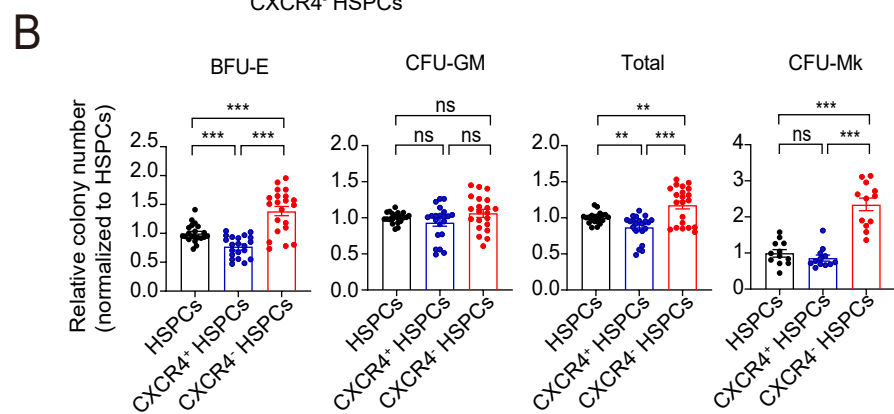

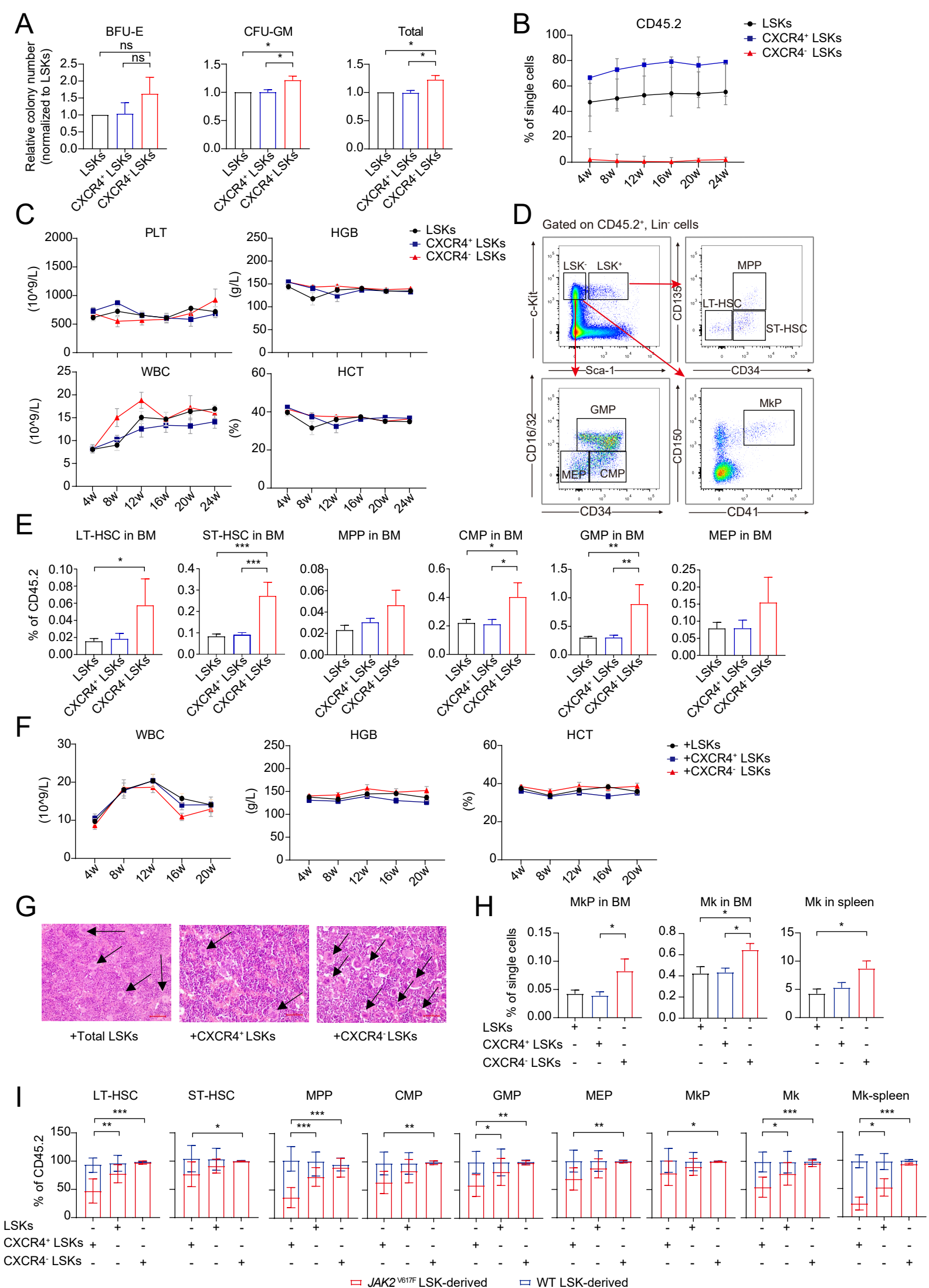

Supplement: Supplementary file 2 — Supporting Information [file ADVS-13-e05249-s003.pdf]
